# Integrating Pharmacists into CGM-Enabled Digital Diabetes Care: Advancing Personalized and Data-Driven Management

**DOI:** 10.3390/healthcare14081019

**Published:** 2026-04-13

**Authors:** Xiaoxiao Chen, Gyeong Eon Kim, Nam Ah Kim, Kwang Joon Kim

**Affiliations:** 1 College of Pharmacy, Chonnam National University, Gwangju 61186, Republic of Korea; vivien250@163.com; 2 College of Pharmacy, Mokpo National University, Muan 58554, Republic of Korea; 3 Department of Biomedicine, Health & Life Convergence Sciences, BK21 Four, Biomedical and Healthcare Research Institute, Mokpo National University, Muan 58554, Republic of Korea

**Keywords:** continuous glucose monitoring, pharmacists, diabetes management, digital health, medication adherence, therapy optimization

## Abstract

**Highlights:**

**What are the main findings?**

- Continuous glucose monitoring (CGM) use is expanding across diabetes populations, yet real-world implementation remains constrained by structural and workflow-related barriers.
- Pharmacist-integrated CGM care supports structured data interpretation and coordinated clinical action, with emerging evidence suggesting potential improvements in glycemic outcomes.

**What are the implications of the main findings?**

- Pharmacists may offer a scalable approach to support CGM-enabled, data-driven diabetes care.
- Integrating pharmacists into CGM workflows may enhance care coordination, responsiveness, and sustained implementation in routine practice.

**Abstract:**

**Background/Objectives**: Continuous glucose monitoring (CGM) has transformed diabetes management by enabling high-resolution assessment of glucose dynamics, with well-established use in type 1 diabetes (T1D) and insulin-treated type 2 diabetes (T2D), and expanding applications across broader populations, including non-insulin-treated T2D and gestational diabetes. However, real-world implementation remains constrained by economic barriers, fragmented reimbursement, workflow challenges, and limited capacity for continuous data interpretation. This review examines key barriers to CGM implementation and synthesizes current evidence on pharmacist-integrated CGM care as an emerging model to support CGM adoption across clinical and community-based settings. **Methods**: A narrative literature review was conducted to synthesize evidence on pharmacist-integrated CGM services in diabetes care. Literature was identified through structured searches of PubMed, Embase, and the Cochrane Library, supplemented by Google Scholar and citation tracking, covering publications from January 2010 to December 2025. Studies were selected based on predefined criteria, including those reporting clinical outcomes, pharmacist involvement, or health system and implementation factors related to CGM use. Relevant survey-based and real-world studies were also considered to capture healthcare professionals’ perspectives and implementation experiences. Evidence was synthesized thematically across clinical, behavioral, and health system domains. **Results**: Available evidence suggests that pharmacist-integrated CGM care is associated with improvements in glycemic management, including increased time in range, reduced glycemic variability, and more timely pharmacotherapy optimization. Pharmacist involvement may also support patient education, self-management, and engagement with digital health technologies, and facilitate ongoing data interpretation and treatment adjustment between clinical encounters. However, evidence remains heterogeneous and geographically limited, with predominantly retrospective and pilot studies and few randomized trials, constraining the robustness and external validity of the findings. Further studies are needed to confirm its clinical effectiveness, comparative effectiveness, and economic value. **Conclusions**: Pharmacist-integrated CGM represents a promising and operationally feasible approach to supporting CGM use in routine diabetes care. While current evidence indicates potential benefits in glycemic management and care delivery processes, further research and implementation efforts are required to support its effective and sustainable adoption across diverse healthcare settings.

## 1. Introduction

Diabetes mellitus (DM) remains a major global public health challenge, with prevalence projected to exceed 783.2 million adults by 2045. Its complications contribute substantially to morbidity, mortality, and economic burden worldwide [1,2,3]. Despite major advances in pharmacotherapy, diabetes outcomes remain suboptimal across many health systems. This is largely attributable to conventional care models that rely on episodic measurements, infrequent clinical encounters, and delayed therapeutic adjustments [4].

Continuous glucose monitoring (CGM) represents a transformative digital technology that provides real-time, high-resolution glucose data, enabling earlier detection of glycemic excursions and more precise therapeutic adjustments [5]. When integrated into digital ecosystems, including mobile health platforms, insulin pumps, and artificial intelligence (AI)-driven analytics, CGM serves as a cornerstone of data-enabled, personalized diabetes care [6,7,8]. Clinical evidence supporting CGM use is strongest in individuals with type 1 diabetes (T1D) and insulin-treated type 2 diabetes (T2D), where improvements in glycemic control and hypoglycemia reduction are well established. Beyond these populations, CGM use is increasingly extending to individuals with non-insulin-treated T2D and diabetes during pregnancy, reflecting a broader shift toward continuous, data-driven glucose monitoring across the diabetes spectrum. However, the clinical benefits of CGM have not translated uniformly into real-world practice. Global uptake remains uneven and is constrained by device costs, fragmented reimbursement policies, regulatory lag, and disparities in digital readiness and workforce capacity. Even in settings where CGM devices are available, patients often receive limited support for data interpretation and behavioral translation. Consequently, high-value glucose data are frequently underutilized. These gaps underscore a fundamental implementation challenge: CGM is not merely a sensing technology but a data-intensive clinical infrastructure that requires continuous interpretation, contextualization, and therapeutic action.

Pharmacists have been increasingly recognized within diabetes care teams as accessible healthcare professionals with expertise in pharmacotherapy, medication optimization, and patient education [9,10]. In community, primary care, and multidisciplinary settings, their expanding scope of practice and sustained patient contact position them to engage with CGM-derived data and support ongoing diabetes management. Within this context, pharmacist involvement has been described in relation to activities such as CGM data interpretation, patient education, and facilitation of treatment adjustments [11]. At the same time, the translation of these roles into measurable clinical and system-level outcomes remains under active investigation. The evidence base for pharmacist-integrated CGM care is marked by substantial methodological heterogeneity, with a predominance of retrospective, pilot, and implementation studies and relatively limited randomized evidence. In this context, pharmacist-integrated models are increasingly being explored as a promising approach to addressing workforce capacity constraints and disparities in access to data-informed care. Available evidence indicates the potential for these models to support improvements in clinical management and care delivery; however, further well-designed and adequately powered studies are required to substantiate their effectiveness and system-level impact. This review synthesizes advances in CGM technology, clinical evidence across the diabetes spectrum, and emerging economic and policy considerations influencing real-world implementation. It further examines the potential contributions of pharmacists within CGM-driven, data-informed diabetes care, while explicitly acknowledging the current limitations of the evidence base.

## 2. Literature Search and Selection

This narrative review was conducted to synthesize evidence on the integration of pharmacists into CGM-enabled diabetes care. A structured literature search was performed in PubMed, Embase, and the Cochrane Library, supplemented by targeted searches in Google Scholar and citation tracking of key publications. Search terms included “continuous glucose monitoring,” “flash glucose monitoring,” “CGM,” and “pharmacist,” with publications considered from January 2010 to December 2025.

Studies were selected based on predefined criteria, including those that (1) reported clinical outcomes of CGM use, (2) involved pharmacist-led or pharmacist-integrated CGM services, or (3) examined health system, reimbursement, or implementation factors. Studies were excluded if they (1) did not involve CGM, (2) did not include pharmacist-related roles or implications, or (3) lacked sufficient detail on outcomes of interest. Study selection involved database retrieval, followed by title and abstract screening and full-text assessment, with inclusion guided by relevance to the review objectives. In total, approximately 20 studies were identified as directly relevant to pharmacist-integrated CGM care, predominantly comprising retrospective, pilot, and implementation studies, with limited randomized evidence.

Survey-based studies, including those assessing healthcare professionals’ perspectives on CGM use and implementation, were also considered where relevant. In addition, reports from governmental agencies, professional organizations, and industry or market research sources were reviewed to provide contextual insights into CGM utilization trends, market expansion, and reimbursement landscapes. These materials were identified through targeted searches of publicly available sources and were included to inform the broader implementation context rather than to support conclusions regarding clinical effectiveness.

## 3. CGM as a Digital Biomarker Enabling Clinical Decision-Making Across Diabetes Populations

CGM has evolved from a supplementary sensing technology into a high-resolution digital biomarker that captures dynamic glucose patterns, treatment effects, and behavioral responses under real-world conditions [12]. Contemporary CGM systems continuously monitor interstitial glucose using enzyme-based electrochemical sensing, most commonly glucose oxidase-mediated reactions coupled with electrode current transduction. Digitized signals are then transmitted to receivers or mobile platforms for real-time visualization and analysis (Figure S1) [13]. Beyond their underlying biochemical and engineering principles, their clinical significance lies primarily in the ability to generate continuous, longitudinal glucose profiles that extend beyond episodic point measurements [14,15,16,17]. Advances in biosensor chemistry, signal calibration, wireless communication, and cloud connectivity have progressively transformed CGM from standalone devices into integrated digital platforms embedded within the broader ecosystem of connected healthcare [18,19,20]. Modern CGM modalities, including professional and personal systems such as real-time CGM (rtCGM) and intermittently scanned CGM (isCGM), now achieve near-reference analytical accuracy, extended wear duration, and seamless interoperability with mobile applications, insulin delivery technologies, and electronic health records (Figure S2) [21,22,23,24]. Increasingly, algorithm-based analytics, including early artificial intelligence-enabled approaches, are incorporated to support proactive interpretation of glucose trends and individualized decision support, further extending the clinical utility of CGM data as a foundation for data-driven, digitally enabled diabetes care [25,26].

Clinically, CGM has transitioned from an adjunctive monitoring tool to a foundational component of data-driven diabetes management across diverse populations. Its clinical utility is most firmly established in individuals with T1D and insulin-treated T2D, where consistent evidence demonstrates improvements in glycemic control and hypoglycemia risk [5,27,28,29,30,31,32] (Tables S2 and S3) and is reflected in current guideline recommendations supporting early and sustained use [33]. Beyond these populations, CGM use is increasingly extending to broader clinical contexts. In non-insulin-treated T2D, emerging evidence supports its role in improving glycemic metrics, facilitating treatment optimization, and enhancing patient engagement [34,35,36,37,38,39,40,41] (Table S3). In pregnancy and gestational diabetes, CGM has been associated with improved glycemic control under conditions requiring tight metabolic regulation [42,43,44,45,46] (Table S4). Taken together, the above evidence highlights the broad clinical contexts in which CGM is applied. The Supplementary tables (Tables S2–S4) summarize representative studies across these settings, serving a contextual and illustrative role rather than constituting efficacy-defining evidence. However, the expansion of CGM use generates substantial clinical demand, as continuous glucose data require ongoing interpretation and timely therapeutic action. These demands are not fully met within existing care models, which remain constrained by limited workforce capacity and episodic care structures. This gap underscores the need for scalable, team-based models to support continuous, data-driven diabetes management.

## 4. Economic, Policy and Equity Considerations Limiting the Real-World Effect of CGM

**Figure 1:**
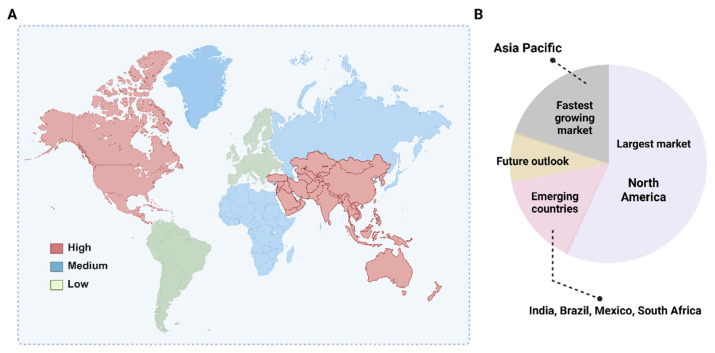
Global variation in CGM market growth and regional dynamics. (**A**) Global distribution of projected CGM market growth categories (high, medium, low) based on relative compound annual growth rates (CAGR). (**B**) Regional CGM market landscape, highlighting North America as the largest market, Asia-Pacific as the fastest-growing region, and emerging markets (e.g., India, Brazil, Mexico, South Africa) with future growth potential. Data source: Mordor Intelligence. Continuous Glucose Monitoring (CGM) Market Size & Share Analysis-Growth Trends and Forecast.

Despite rapid technological advancement, the adoption of CGM remains uneven globally, constrained by market dynamics, reimbursement variability, and systemic barriers to equitable access. The CGM market has expanded rapidly within a highly concentrated industry landscape, where a small number of manufacturers shape innovation trajectories, pricing structures, and global diffusion [15]. Market growth is strongest in regions with mature reimbursement and digital health infrastructure, especially North America, whereas Europe shows steady adoption while Asia-Pacific grows fastest, driven by ageing population, rising diabetes prevalence, and health-system modernization [47] (Figure 1). Technological innovation has increasingly focused on extended-wear sensors and cloud-connected platforms, supporting a transition toward decentralized, home-based monitoring. In such settings, CGM data are primarily used for self-management and behavioral decision-making, with the potential for remote sharing and clinician input where appropriate digital infrastructure is available.

Despite rapid technological advances, CGM remains cost-intensive. High cost of sensors, transmitters, and proprietary data platforms limits access in low- and middle-income countries and sustains substantial out-of-pocket spending in high-income health systems. Subscription models may reduce upfront costs but often remain inaccessible for many users. Innovative pay-per-use or outcomes-based contracts may reduce financial hurdles, but broad implementation remains limited. Beyond device costs, the total economic burden of CGM includes data infrastructure, clinical interpretation, and patient education. These components are central to the value of CGM but are seldom covered by current reimbursement schemes. Consequently, patients receive CGM devices without sufficient support to translate glycemic data into actionable decisions, limiting real-world effectiveness and exacerbating inequities in diabetes management [47]. Reimbursement policies for CGM vary substantially across regions, reflecting differences in health system structures, payer priorities, and evidentiary thresholds (Table 1) [48]. Several countries, including Canada, the United Kingdom, France, Korea, and Australia, provide relatively broad public coverage for individuals with T1D and selected high-risk populations with T2D [49]. In contrast, reimbursement in large markets such as the United States remains fragmented across Medicare, Medicaid, and commercial insurance systems, resulting in inconsistent access even among clinically eligible individuals. Many policies continue to rely on restrictive or outdated eligibility criteria, such as requirements for multiple daily insulin injections or frequent self-monitoring of blood glucose. Furthermore, reimbursement is typically limited to device provision, with insufficient coverage for essential services such as data interpretation, patient education, and follow-up [50].

In many health-care systems, coverage policies lag behind technological innovation, with payers deferring reimbursement until long-term cost-effectiveness data—including reduced complications or hospitalizations—are available rather than recognizing intermediate glycemic and quality-of-life benefits [51]. These delays are particularly pronounced in low-resource settings, where public reimbursement is limited or absent and out-of-pocket costs remain prohibitive [51,52]. Moreover, misaligned incentives across stakeholders further constrain implementation: insurers prioritize short-term cost containment; providers face increased workload without additional reimbursement for CGM interpretation; and patients—especially from underserved populations-navigate complex preauthorization, renewal, and copayment processes [52,53]. These structural barriers contribute to persistent inequities in CGM access and utilization. Lower rates of CGM prescription and use have been reported among racial and ethnic minorities, uninsured individuals, and socioeconomically disadvantaged populations, as well as among those receiving care in resource-limited settings such as rural areas or federally qualified health centers [54,55]. Even when access is achieved, the absence of reimbursement for data review, patient education, and follow-up limits the effective use of CGM data and reduces its potential population-level impact [53]. Overall, the uneven diffusion of CGM reflects not only financial constraints but also broader systemic limitations in digital readiness, workforce capacity, and care delivery infrastructure. Addressing these challenges will require rethinking CGM-enabled care models and integrating digital technologies into coordinated, team-based workflows that extend beyond traditional physician-centric approaches.

**Table 1:** Global variation in CGM reimbursement policies across major health systems.

Country	Reimbursement/Coverage	Primary Eligibility Criteria *
Canada [56]	Full public coverage for isCGM/rtCGM; limited short-term coverage during pregnancy	T1D; insulin-treated T2D; high-risk populations (e.g., hypoglycemia risk, safety-sensitive occupations); pregnancy requiring insulin
United Kingdom [49,57]	NHS funding for CGM/isCGM under national/local commissioning criteria	T1D (routine coverage); selected T2D populations and other high-risk groups per NHS eligibility criteria
USA [39]	Medicare and private insurers reimburse therapeutic CGM for insulin-treated patients; Medicaid coverage varies by state, while private coverage varies across insurers and employer-sponsored benefit designs.	Insulin-treated diabetes under Medicare tCGM criteria; additional groups eligible under state-specific policies.
Germany [58]	Statutory health insurance reimburses CGM for eligible patients following G-BA/SSV decisions.	Patients using insulin therapy and defined high-risk subgroups meeting G-BA eligibility criteria.
Japan [59]	National health insurance reimbursement	As of December 2022, coverage was expanded to include all individuals self-injecting insulin ≥1 daily, regardless of diabetes type
South Korea [60,61,62]	National health insurance reimbursement	Primarily T1D; expanding coverage for insulin-treated T2D
France [58]	National reimbursement for approved CGM systems	Individuals using intensive insulin therapy; expanding eligibility for selected T2D groups, including non-intensively treated patients (>3 injections/day) with inadequate glycemic control)
Spain [63,64]	Decentralized reimbursement decisions at the regional (autonomous community) level	T1D and selected T2D populations, eligibility varies based on regional HTA decisions and budget priorities
Australia [65]	Subsidized access through the NDSS	All individuals with T1D; children, insulin-treated individuals, and other NDSS-defined high-need groups meeting program criteria

* Eligibility criteria may vary across regions, insurance systems, and over time. Notes: Policy information reflects publicly available sources up to October 2025. Data were synthesized from national reimbursement policies, government or health system websites, and relevant literature. Reimbursement criteria and eligibility are dynamic and may vary within countries depending on regional policies, payer structures, and updates to clinical or policy guidance. Abbreviations: CGM, continuous glucose monitoring; isCGM, intermittently scanned CGM; rtCGM, real-time CGM; T1D, type 1 diabetes; T2D, type 2 diabetes; NHS, National Health Service; G-BA, Federal Joint Committee (Gemeinsamer Bundesausschuss); HTA, health technology assessment; NDSS, National Diabetes Services Scheme.

## 5. Pharmacist-Enabled Implementation of CGM in Digitally Enabled Diabetes Care

The implementation of CGM in routine diabetes care can be conceptualized as a multi-level, digitally enabled system comprising three interdependent components: data infrastructure, healthcare workforce capacity, and coordinated care pathways. CGM devices and digital platforms enable the continuous generation, transmission, and visualization of high-frequency glucose data, while pharmacists facilitate the distributed interpretation of CGM-derived information and supports data-informed therapeutic decision-making. Coordinated care pathways translate these data into timely clinical actions through structured, collaborative workflows.

As CGM expands beyond specialist settings and insulin-treated populations, traditional physician-centric care models are increasingly misaligned with the demands of continuous data review and iterative therapeutic adjustment. Conventional care models limit the translation of high-frequency glucose data into timely interventions, creating a fundamental implementation gap. In this context, pharmacists are well positioned to bridge data generation and clinical action. Their accessibility, pharmacotherapeutic expertise, and expanding role in chronic disease management enable them to function as key intermediaries within digitally enabled CGM care. Importantly, pharmacist-integrated CGM care is not envisioned as brief, opportunistic over-the-counter counseling, but rather as a structured, appointment-based clinical service delivered longitudinally and integrated within defined diabetes care pathways. Against this background, pharmacists are increasingly being repositioned as key actors in translating CGM data into actionable clinical care.

### 5.1. Community Pharmacist-Integrated CGM Interpretation and Patient Engagement

Within this framework, community pharmacists are increasingly positioned as distributed interpreters of CGM data, helping to address the gap between continuous data generation and actionable clinical decision-making. Compared with specialist-centered models, community pharmacies provide frequent patient contact and sustained longitudinal relationships, facilitating repeated cycles of data review, education, and follow-up [66,67]. In practice, pharmacist-integrated CGM services are generally delivered through structured consultations at defined clinical touchpoints.

In settings with limited access to specialist care, community pharmacists may serve as accessible intermediaries linking CGM technologies with day-to-day diabetes management. Evidence suggests that CGM may offer clinical benefits beyond insulin-treated populations, including in non-insulin-treated T2D when combined with structured interpretation and feedback. However, such populations remain underrepresented in specialist-led CGM programs [9,68]. In this context, community pharmacist-integrated models represent a potential extension of CGM-enabled care into routine, non-specialist settings. Although community pharmacy-specific evidence remains limited, as most studies are conducted in ambulatory or clinic-based environments, available data suggest that pharmacist involvement is associated with improved glycemic outcomes, patient empowerment, and diabetes-related quality of life [69]. This limited evidence base may partly reflect implementation challenges, including competing responsibilities, limited protected time, and the complexity of integrating CGM workflows into routine pharmacy practice.

A key contribution of community pharmacists lies in integrating CGM-derived metrics with medication use, diet, daily routines, and adherence during routine patient encounters [66]. This continuous, context-aware interpretation enables iterative, behaviorally informed interventions that are challenging to achieve through episodic specialist visits alone. For example, in a federally qualified health center (FQHC)-based pharmacist-integrated CGM program, patients were referred through provider referral or shared-visit models and received a scheduled, one-on-one, face-to-face initial appointment of approximately 60 min with a clinical pharmacist. This visit focused on CGM education alongside comprehensive review of medications, adherence, affordability, diet, physical activity, and comorbid conditions [70]. Patients subsequently attended planned follow-up visits of approximately 30 min dedicated to CGM data upload, structured report interpretation, and therapy optimization. This model demonstrates the feasibility of structured, longitudinal pharmacist-integrated CGM care. Emerging prospective studies of community pharmacist-led remote CGM monitoring further support this model, showing clinically meaningful improvements in metrics including time in range and mean glucose [69]. In these models, remote interactions may be enabled through cloud-connected CGM platforms that allow scheduled or asynchronous review of longitudinal glucose profiles, with pharmacists focusing on pattern interpretation in relation to medication regimens, dosing timing, adherence, and daily routines [69]. Beyond glycemic outcomes, these models may improve access to structured data interpretation in community settings, particularly for populations with limited specialist access or lower digital literacy [67]. Importantly, these pharmacist-integrated remote CGM activities are designed to complement rather than replace physician and diabetes nurse educator roles by assuming responsibility for routine, data-intensive CGM review, while maintaining clear escalation pathways for complex clinical decision-making or intensive diabetes education. Nevertheless, broader implementation depends on regulatory scope, reimbursement for cognitive services, digital interoperability, and integration within multidisciplinary care pathways. Addressing these system-level constraints will be essential to realizing the full translational impact of CGM in routine, population-level diabetes care.

### 5.2. Clinical Pharmacist Integration in Multidisciplinary CGM-Enabled Diabetes Care

In hospital-based and ambulatory care teams, clinical pharmacists play a central role in managing complex pharmacotherapy, optimizing treatment intensification and supporting medication safety. Integrating CGM into these settings further amplifies pharmacist expertise, especially for patients with advanced disease, multimorbidity, or higher hypoglycemia risk [67]. Clinical pharmacists integrate CGM metrics with medication regimens, renal function, comorbidities, and patient-reported outcomes to guide individualized therapeutic decisions, including insulin titration, deprescribing, or transitions to novel glucose-lowering agents. Available evidence, primarily from retrospective and real-world studies, suggests that clinical pharmacist-assisted CGM initiation and follow-up are associated with reductions in HbA1c and improvements in CGM-derived metrics across diverse patient cohorts [71,72]. Integrated programs incorporating pharmacists into CGM workflows report improved glycemic control, increased time in range (TIR), and enhanced clinical outcomes compared to usual care. In a large tertiary health system, pharmacist-integrated CGM management was associated with significant HbA1c reduction and improved CGM metrics at 3 and 6 months [73]. Where reimbursement mechanisms are available, pharmacist-integrated CGM services are typically supported through billing for structured CGM data interpretation and cognitive clinical services—such as report analysis, therapy optimization, and care coordination under collaborative practice agreements—rather than routine medication dispensing activities. These models highlight the evolving role of pharmacists as digital custodians, promoting data continuity, adherence to evidence-based objectives, and timely therapeutic interventions in data-driven environments. However, the effective implementation of these roles depends on structured training, competency development, and familiarity with CGM data interpretation and digital health tools, which remain variable across healthcare settings, highlighting an important implementation gap that may influence the scalability of pharmacist-integrated CGM care. Collectively, these evolving roles position pharmacists as key enablers of the clinical and societal value of CGM. Embedding CGM interpretation within pharmacist-integrated and integrated care models may help health systems address economic, policy, and equity constraints, transforming CGM from a device-centric innovation into a scalable, data-driven intervention that supports sustainable and equitable diabetes management.

### 5.3. Pharmacist-Coordinated Interprofessional CGM Care Enabled by Patient-Generated Data

**Figure 2:**
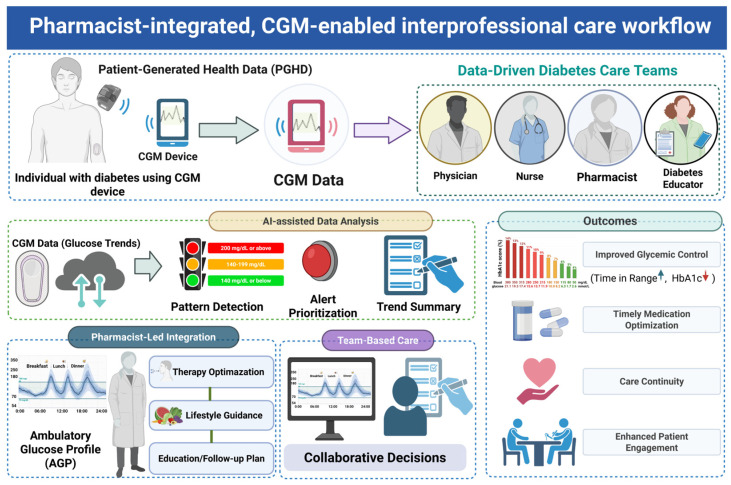
Pharmacist-integrated, CGM-enabled interprofessional care workflow. CGM generates patient-generated health data (PGHD), which are transmitted through digital platforms and interpreted using structured workflows, including pattern recognition, alert prioritization, and trend summarization. Pharmacists integrate CGM-derived metrics with medication use, lifestyle factors, and clinical context to support therapeutic optimization, patient education, and follow-up planning. Within interprofessional care teams, these pharmacist-integrated processes inform collaborative clinical decision-making involving physicians, nurses, and diabetes educators. The workflow illustrates a data-informed care model that supports coordinated interpretation and application of CGM data in routine practice, with potential relevance for glycemic management, medication optimization, continuity of care, and patient engagement. (Created with BioRender.com).

CGM data are a type of patient-generated health data (PGHD) that provides a shared, longitudinal, and actionable information foundation for interprofessional diabetes care [10,74,75] (Figure 2). These data are typically accessed through CGM-related digital applications, including cloud-based platforms, mobile interfaces, and remote monitoring dashboards, which enable real-time data sharing and longitudinal glucose assessment across care teams. Within CGM-enabled service models, pharmacists frequently coordinate the longitudinal review and synthesis of CGM-derived PGHD, acting as a central coordinating node in interprofessional workflows [66,69]. After CGM initiation, glucose data are transmitted to pharmacists through digital platforms, where CGM metrics are integrated with medications, lifestyle factors, and relevant clinical context [72]. Using structured protocols, pharmacists translate high-frequency glucose data into clinically actionable signals for medication adjustment, self-management, and follow-up. These interpretations trigger downstream clinical actions without duplicating physician decision-making and support task distribution across care teams through clearly defined communication pathways linking pharmacists, primary care physicians, and diabetes educators. Pharmacist-initiated, CGM-informed recommendations may be reinforced by nurses and diabetes educators through behavioral counseling and adherence support with escalation pathways ensuring timely physician involvement when risks or treatment failure arise [76]. Effective participation in these workflows requires pharmacists to possess competencies in CGM data interpretation and proficiency in CGM-related digital applications, including familiarity with data visualization tools, remote monitoring systems, and clinical decision-support interfaces. These requirements highlight the importance of structured training to support consistent and scalable implementation.

In specialist-led care settings, particularly for individuals with T1D or complex insulin-treated T2D, endocrinologists typically serve as the primary prescribers and maintain longitudinal responsibility for treatment decisions [77]. Within this context, pharmacist-integrated CGM workflows complement specialist care by supporting continuous data interpretation, identifying clinically relevant glycemic patterns, and facilitating timely communication of actionable insights [78]. Pharmacists may also contribute to interim management through protocol-informed adjustments, patient education, and follow-up between clinical encounters, while maintaining appropriate physician oversight [78]. Positioned between patients, primary care, and specialty services, pharmacists maintain longitudinal CGM oversight to ensure coherent therapeutic decision-making across care transitions. This continuity enhances accountability and mitigates therapeutic inertia and care fragmentation, particularly in patients with complex regimens or high glycemic variability.

From a health system perspective, the role of pharmacists varies according to the availability of specialist care. In settings with limited access to endocrinologists or specialist diabetes services, pharmacist-integrated CGM models may help mitigate workforce constraints by supporting data-informed management and facilitating timely escalation of care [79]. In contrast, in settings where specialist resources are more readily available, pharmacists may function as care extenders within multidisciplinary teams, enabling more frequent CGM data review, accommodating higher patient volumes, and strengthening coordination across primary and specialty care providers [77]. Across both contexts, pharmacist integration enhances the responsiveness, scalability, and continuity of CGM-enabled diabetes care, while reinforcing the complementary roles of pharmacists and physicians within coordinated, data-driven care models.

## 6. Clinical Evidence Supporting Pharmacist-Integrated CGM Services

Current evidence for pharmacist-integrated CGM services is methodologically heterogeneous and largely dominated by retrospective, single-arm, pilot, and implementation studies, with limited RCTs. Growing evidence highlights the expanding role of pharmacist-integrated CGM interpretation in supporting glycemic management, therapeutic optimization, and patient-centered outcomes.

Pharmacist integration into CGM-driven diabetes care has been reported across community, ambulatory, and academic care settings, with studies indicating potential clinical and operational feasibility [10]. Observational and prospective studies report short-term reductions in HbA1c and improvements in CGM-derived metrics following structured pharmacist-integrated interventions (Table 2). In a large multicenter academic health system, pharmacist-managed CGM review implemented within routine care was associated with mean HbA1c reductions of 1.48% at 3 months and 1.74% at 6 months, alongside a 12% increase in TIR, across a heterogeneous and clinically complex population of adults with T2D [73]. Community pharmacy and pilot clinic studies similarly report sustained TIR gains, reduced glycemic variability, and improved adherence after structured pharmacist interpretation and follow-up [69,80]. Real-world evaluations further indicate that pharmacist-integrated CGM models are operationally feasible across diverse care settings. Retrospective analyses of community pharmacy-based CGM services have reported mean HbA1c reductions of approximately 1.2% over 3 months, accompanied by high patient satisfaction and willingness to continue CGM-supported care [67]. Implementation studies also suggest that pharmacist-integrated CGM services can be incorporated into existing clinical workflows and delivered through collaborative practice models, enabling pharmacists to contribute to CGM-related care alongside physicians while leveraging established reimbursement pathways [78]. However, the current evidence is limited by small sample sizes, short follow-up durations (typically 3–6 months), and single-center or highly structured study settings. These limitations constrain generalizability and underscore the need for larger, multicenter studies with longer follow-up to more robustly evaluate the effectiveness, scalability, and sustainability of pharmacist-integrated CGM services.

## 7. Health System and Economic Considerations of Pharmacist-Integrated CGM Care

Emerging evidence suggests that pharmacist-integrated CGM services may be associated with economic and system-level implications beyond glycemic outcomes, although current data remain limited and context-specific. A recent model-based cost-effectiveness analysis found that pharmacist-led care was associated with improved clinical outcomes and reduced overall costs, representing a dominant strategy with an estimated cost reduction of approximately $1469 per patient over the modeled time horizon [87]. While these findings provide preliminary signals of potential economic value, they are derived from modeled assumptions and should be interpreted with caution. In the United States, a CGM pilot in a federally qualified health center reported a 1.95% reduction in HbA1c and approximately $5979 in annual reimbursement per patient through structured CGM interpretation (CPT 95251), demonstrating operational feasibility and potential revenue generation [80]. Similarly, a community pharmacy-based CGM service reported annualized revenues of USD7052 per pharmacist, suggesting that CGM services may generate meaningful revenue within existing pharmacy practice frameworks, although longer-term evidence is needed to confirm sustainability [78]. Broader evaluations of pharmacist-managed chronic disease programs report per-patient cost savings ranging from USD7 to USD65,000 and benefit-to-cost ratios of up to 8.5:1. While not specific to CGM-integrated care, these findings suggest a potential economic rationale for pharmacist-involved care models [88].

Beyond economic considerations, pharmacist involvement in CGM-enabled care has been described in relation to care delivery processes and system capacity. Pharmacists are widely distributed across ambulatory and community settings, and their integration into care teams has been associated with roles in CGM initiation, patient education, data interpretation, and follow-up. These functions may help support access to CGM-enabled care and facilitate ongoing data interpretation within routine practice. Real-world data also suggest that factors such as acquisition channels and adherence patterns may influence both outcomes and healthcare utilization. For example, differences in adherence and costs have been observed between durable medical equipment and pharmacy-based CGM supply pathways [89]. These findings highlight the complexity of care delivery pathways and suggest that system-level outcomes are shaped by multiple interacting factors beyond any single care model.

Within this broader context, pharmacist-integrated CGM services may contribute to addressing a practical gap in care delivery by supporting the interpretation and application of CGM data in routine practice. Through activities such as data review, patient education, and coordination within care teams, pharmacists may facilitate the translation of CGM-derived information into clinical decision-making [10]. Importantly, most reported pharmacist-integrated CGM programs do not rely on dedicated, standalone pharmacist full-time equivalents (FTEs), but instead incorporate CGM-related activities into existing clinical roles, with staffing intensity adapted to patient volume and care complexity rather than fixed FTE thresholds. However, given the current limitations of the evidence base, including heterogeneity in study design and reported outcomes, the effectiveness, scalability, and system-level impact of these models remain to be established through further well-designed studies.

## 8. Implementation Pathways and Future Directions for Pharmacist-Integrated CGM Services

Adoption of pharmacist-integrated CGM services within contemporary health systems will depend less on further technological advances than on alignment of regulatory scope of practice, reimbursement for cognitive clinical services, and digital interoperability. Health systems that formally integrate pharmacists into CGM interpretation and longitudinal diabetes management pathways may be better positioned to achieve scalable and sustainable implementation of CGM-enabled care. Importantly, the expansion of CGM-enabled care is occurring against a backdrop of limited and, in many regions, declining access to specialty diabetes care. Workforce analyses indicate a persistent global shortage of endocrinologists, with maldistribution favoring urban tertiary centers and widening gaps in rural and underserved areas [90,91,92]. In the United States, fewer than half of counties have a practicing endocrinologist, and wait times for specialty diabetes care frequently exceed several months [91]. Similar constraints have been reported across Europe and low- and middle-income countries, where specialist capacity remains insufficient to meet the growing burden of diabetes [90]. Within this context, pharmacist-integrated CGM models may provide a practical mechanism for redistributing interpretive and therapeutic tasks traditionally concentrated in specialist care. Central to this model is the structured use of standardized AGP reports to support pharmacotherapy assessment. Through systematic interpretation of AGP patterns in relation to medication class, dosing schedules, and expected pharmacodynamic profiles, pharmacists can help identify whether glycemic abnormalities result from inadequate basal coverage, insufficient postprandial control, suboptimal dosing timing, or adherence-related factors. This AGP-pharmacotherapy alignment enables more targeted identification of residual treatment gaps and supports tailored medication adjustments and patient education within existing care pathways.

Future expansion will require investment in interoperable data systems and standardized workforce training to support consistent CGM interpretation across care settings. Such training should extend beyond device-specific competencies to include advanced clinical skills in diabetes management, potentially supported by formal certification pathways (e.g., Board Certified-Advanced Diabetes Management or Diabetes Care and Education Specialist (CDCES) credentials) [93]. Community and ambulatory pharmacies, among the most accessible points of care in many health systems, offer a practical platform for extending CGM-enabled services beyond specialist settings, particularly in regions with limited physician availability [94,95]. To address variability in resources and equity considerations, adaptable service models—including professional and intermittently scanned CGM—will be essential. Looking ahead, pharmacist-integrated CGM services are expected to evolve from episodic data review toward more continuous and coordinated models of diabetes management. Integration of CGM data with electronic prescribing, adherence monitoring, and population health analytics may enable more proactive identification of risk and stratified intervention. Collectively, these developments suggest that pharmacist-integrated CGM services may represent a scalable approach to supporting coordinated, data-informed, and equitable diabetes care, although further evidence is required to establish their system-level impact.

## 9. Discussion

Pharmacist-integrated CGM care is emerging alongside rapid technological advances and expanding access beyond specialist settings. As CGM systems become increasingly user-friendly and accessible in community settings, pharmacists are well positioned to support device selection, patient education, data interpretation, and longitudinal follow-up. This evolving role aligns with broader shifts toward data-driven, patient-centered and interdisciplinary care models. Accumulating evidence indicates that pharmacist involvement in CGM workflows is associated with improved glycemic control, reduced hypoglycemic events, and more individualized diabetes management across diverse care settings [70].

Importantly, the clinical application of pharmacist-integrated CGM care differs across diabetes populations. In T1D and insulin-treated T2D, where CGM-derived metrics such as TIR and glycemic variability are well established, CGM is primarily used to guide therapeutic adjustments, support hypoglycemia prevention, and inform day-to-day management of glycemic patterns. In these settings, pharmacist involvement focuses on the structured interpretation of CGM data to inform individualized treatment decisions, including insulin titration and optimization of pharmacotherapy. By contrast, in non-insulin-treated T2D, CGM is more often applied to support behavioral modification, enhance patient engagement, and facilitate early identification of dysglycemia. In other populations, including gestational diabetes, CGM is primarily used to support closer glycemic monitoring and timely therapeutic adjustment to achieve strict glycemic targets, reflecting distinct clinical priorities and decision-making pathways.

The value of pharmacist-integrated CGM care lies not only in access to continuous glucose data, but in its structured, pharmacotherapy-focused interpretation. Pharmacists are uniquely positioned to contextualize CGM-derived glucose patterns within individualized treatment regimens, supported by standardized tools such as AGP. However, the current evidence base is methodologically heterogeneous and geographically concentrated, with most studies conducted in the United States. Most studies consist of retrospective analyses, single-arm or pilot studies, and implementation evaluations, with relatively few randomized controlled trials. This heterogeneity, along with modest sample sizes and relatively short follow-up periods, limits definitive conclusions regarding comparative effectiveness and broad generalizability of pharmacist-integrated CGM services across healthcare systems. Notably, several studies reporting larger effect sizes were conducted in underserved or resource-constrained settings. In these contexts, baseline therapeutic gaps and workforce limitations may amplify the observed impact of pharmacist-integrated care. While these findings suggest potential contextual value, their applicability across diverse healthcare systems remains uncertain. Further validation in larger, well-designed prospective studies is therefore required.

Despite early evidence of clinical benefit, pharmacist-integrated CGM services face substantial barriers that limit their scalability and long-term sustainability [96]. Emerging survey-based evidence, including studies among primary care providers and pharmacy trainees, provides complementary insight into real-world implementation, indicating generally positive attitudes toward CGM among healthcare professionals and trainees, while highlighting persistent challenges related to workforce capacity, workflow integration, and health system support [97,98]. Educational and training gaps are a key constraint, as proficiency with evolving CGM technology, apps, customizable alerts, and data integration into personalized care often requires learning beyond traditional curricula [99]. In parallel, workflow-related challenges, such as competing clinical responsibilities, limited protected time, and the complexity of incorporating CGM data into routine care processes, further hinder implementation. Structural barriers, including inconsistent reimbursement, variable payer coverage, and regulatory and interoperability limitations, also constrain broader adoption. Beyond these implementation challenges, potential limitations and unintended consequences of pharmacist-integrated CGM models warrant careful consideration [97]. Variability in pharmacist training and experience may lead to inconsistent clinical decision-making in the absence of standardized competency frameworks. Furthermore, the continuous generation of high-volume CGM data may contribute to information overload and alert fatigue if not supported by structured interpretation protocols and decision-support systems. Emerging digital tools, including AI-enabled analytics, may help address some of these challenges. These tools support pattern recognition, predictive modeling, and early identification of high-risk glycemic events, enabling more proactive and personalized diabetes management [100,101,102]. These approaches can assist pharmacists and other healthcare providers by identifying high-risk glucose patterns, supporting triage decisions, and reducing cognitive burden associated with longitudinal CGM data review [101,103]. Within this framework, AI functions as an enabling technology that extends the reach and efficiency of pharmacist-integrated CGM services while preserving clinical oversight, contextual judgment, and patient-centered decision-making. However, current evidence remains insufficient to support routine implementation. At present, AI-assisted CGM workflows should be considered exploratory rather than standard practice.

Building on these considerations, future work should prioritize the translation of pharmacist-integrated CGM care from descriptive evaluation to structured implementation, supported by large-scale, well-designed randomized controlled trials to establish clinical effectiveness and cost-effectiveness across diverse patient populations and care settings. A key priority is the establishment of clearly defined competency domains for pharmacists, encompassing the interpretation of CGM metrics and AGP reports, integration of digital tools into clinical workflows, recognition of clinically actionable glycemic patterns, and delivery of tailored patient education. Embedding these competencies within standardized training and certification pathways will be essential to ensure consistency and safety in practice. Equally important is the development of scalable care delivery models that integrate CGM into routine clinical workflows across settings, including pharmacist-led services, collaborative ambulatory care models, and telehealth-enabled monitoring systems. In addition, implementation research in low- and middle-income countries is needed to evaluate the feasibility, scalability, and context-specific impact of pharmacist-integrated CGM services under resource-constrained conditions. In parallel, there is a need for pragmatic clinical frameworks that specify decision-making roles and escalation pathways in CGM-informed care, particularly for time-sensitive events such as hypoglycemia. Clarifying the boundaries between protocol-driven pharmacist autonomy and physician collaboration will be critical to supporting safe, efficient, and sustainable care delivery within diverse healthcare systems. Collectively, these priorities underscore the need for an integrated, evidence-based implementation framework that aligns technological innovation with workforce capacity and health system readiness, thereby enabling the scalable and equitable adoption of CGM-enabled, pharmacist-integrated diabetes care.

## 10. Conclusions

CGM has emerged as a central component of personalized and data-driven diabetes care, enabling continuous assessment of glycemic patterns and more responsive therapeutic management. Integrating pharmacists into CGM-enabled care models represents an evolving approach to supporting patient education, facilitating therapy optimization, and sustaining longitudinal diabetes management across care settings. However, access to CGM remains uneven, particularly among individuals with T2D, reflecting broader economic, policy, and health system constraints. The current evidence base for pharmacist-integrated CGM care is characterized by methodological heterogeneity and limited randomized data. Further well-designed, large-scale randomized trials and prospective real-world studies are needed to clarify the clinical effectiveness, scalability, and economic implications of these models. Addressing these challenges will require not only strengthening the evidence base but also aligning reimbursement policies, reducing administrative barriers, and supporting the integration of pharmacists within multidisciplinary, digitally enabled care pathways. Such efforts may facilitate more equitable access to CGM and support its translation from technological innovation into routine, system-level diabetes care.

## Figures and Tables

**Table 2:** Representative clinical studies evaluating the clinical and implementation outcomes of pharmacist-integrated CGM services in diabetes care.

Study (First Author, Year)	Study Design	Care Setting	Population (n)	CGM Type	Intervention	Comparator	Baseline	Follow-Up	Primary Outcomes	Key Findings
Van Dril, 2019 [81]	Retrospective study	Outpatient ambulatory care	Adults with T1D or T2D (n = 29)	Pro CGM	Clinical pharmacist-managed Pro CGM	N/A	HbA1c: 9.0 ± 1.7%	N/A	HbA1c: −0.7 ± 2.0%	Reduced HbA1c and facilitated patient behavioral changes with pharmacist-guided CGM care
Sherrill, 2020 [10]	Retrospective comparative study	Outpatient clinic	Patients who had a CPT code 95250 or 95251 (CGM-specific billing codes) (n = 378)	Pro CGM	Pharmacist or physician-managed Pro CGM	Pharmacist vs. physician	HbA1c: RPh1: 8.4 ± 1.5%; RPh2: 9.0 ± 1.8%; MD1: 9.2 ± 1.6%	6 months	HbA1c: −1.0% (1 RPh); −1.3% (2 RPh visits); −0.6% (MD1)	Greater HbA1c reduction and more pharmacologic interventions were observed with pharmacist-driven Pro CGM compared with physician-led care
Ulrich, 2021 [82]	Retrospective study	Ambulatory care clinic	Adults with T2D (n = 30)	Pro CGM	Clinical pharmacist-managed Pro CGM	N/A	HbA1c: 9.1 ± 1.4%	6 months	HbA1c: −0.47% (3 months); −0.92% (6 months)	Pharmacists employing Pro CGM as part of a diabetes management service were able to improve glycemic control for patients with T2D.
Simonyan, 2021 [83]	Retrospective study	Outpatient endocrinology or primary care clinics	Adults with T1D, T2D, or prediabetes (n = 171)	Pro CGM	Combines DSMES and Pro CGM into a shared medical appointment (SMA) format	N/A	HbA1c: 8.64 ± 1.99%	N/A	HbA1c: −0.80 ± 1.52%; Diabetes self-efficacy score: +1.63 ± 2.09 points from baseline (6.56 ± 2.61)	The CGM SMA is a novel practice model incorporating diabetes education, pharmacist, Pro CGM, and interprofessional care that demonstrated improvements in diabetes self-efficacy and A1C.
Fantasia, 2021 [84]	Clinical pilot program	Ambulatory care setting (clinical pilot)	Adults with T2D (n = 74)	Pro CGM	Endocrinology or clinical pharmacists-manage d Pro CGM	Endocrinology vs. clinical pharmacists	HbA1c: Endocrinology: 10.5 + 2.5%; pharmacist: 10.2 + 2.0%	3 months	HbA1c: Endocrinology: −1.0 ± 2.0%; pharmacist: −1.6 ± 1.8%	Similar short-term glycemic outcomes with CGM-enhanced eConsults and in-person visits, with good acceptability among pharmacists and patients
Powell, 2024 [85]	Observational report	Outpatient clinic serving underserved populations	Underserved adults with T2D (n = 41)	N/A	Pharmacist-managed CGM	Pharmacist visit vs. No pharmacist visits	HbA1c: 10 ± 2.5%	18 months	HbA1c: Pharmacist visit: −2.5 ± 2.7%; No pharmacist visits: −0.8 ± 1.6%	Multidisciplinary care including pharmacists may improve outcomes, with noted barriers to CGM access and care delivery
Pasour, 2024 [78]	Prospective, investigator-initiated pilot study	Atrium Health Internal Medicine clinic	Adults with diabetes (n = 42; 30 completed)	Personal CGM	Pharmacist-led personal CGM workflow	Pre-CGM Workflow vs. Post-CGM Workflow	HbA1c: 8.3%	28 weeks	HbA1c: −2.1%	Pharmacist-led personal CGM workflow was associated with improved care quality, with a potential reduction in hospitalizations requiring further confirmation
Ballard, 2024 [80]	Prospective pilot study	FQHC	Adults with diabetes (n = 15)	No exceptions to brand of CGM device	Pharmacist-led CGM clinic (with no exceptions to brand of CGM device)	N/A	HbA1c: 10 ± 2.49%	N/A	HbA1c: −1.95%; TIR: +9.2%	Pharmacist-led CGM clinic may have a positive impact on clinical and financial outcomes within a FQHC
Beldon, 2024 [69]	Prospective feasibility study	Chain community pharmacies	Adults with diabetes (n = 36)	Remote CGM	Community pharmacist-led enrollment and remote CGM monitoring through cloud-based platforms	N/A	HbA1c: 7.59%; Very high TAR: 14.2 ± 18.95%; High TAR: 22.6 ± 9.38%; TIR: 61.8 ± 25.89%; Low TBR: 0.9 ± 2.21%; Very low TBR: 0.5 ± 2.01%	3 months	Very high TAR: 7.95 ± 10.53%; High TAR: 21.2 ± 10.56%; TIR: 69.9 ± 19.57% Low TBR: 0.9 ± 1.89%; Very low TBR: 0.05 ± 0.22%	Community pharmacists-led remote CGM monitoring with improvements in glycemic metrics and support for medication and behavioral management
Miller, 2024 [67]	Program evaluation	Community and inpatient pharmacy services	Adults with diabetes (n = 18)	Pro CGM	Community-based Pharmacy resident-driven CGM service	N/A	HbA1c: 9.7%	3 months	HbA1c: −1.2%; Forty CPT codes were billed, generating $3671.40 of revenue	Community-based pharmacy resident-driven implementation of CGM can produce cohort reductions in HbA1c and generate revenue for the clinic and pharmacy.
Mnatzaganian, 2025 [73]	Retrospective cohort study	Ambulatory care clinics	Adults with T2D (n = 165)	Personal CGM	Pharmacist-led Diabetes Management and Education Clinic (DMEC) on patients with T2D using a personal CGM	N/A	HbA1c: 9.15 ± 1.9%	2 years	HbA1c: −1.48% (3 months), −1.74% (6 months); TIR: +8.2% (3 months), +12.1% (6 months)	Pharmacist-led management of T2D using personal CGMs can improve diabetes outcomes in a large academic health care system.
King, 2025 [72]	Retrospective study	Outpatient clinics	Adults with T2D (n = 317)	Personal CGM	Pharmacist involvement in personal CGM management	Pharmacist-led management vs. the usual care	N/A	N/A	Percentage of patients de-prescribed a high-risk medication: Pharmacist-led intervention (11.4%) vs. usual care (8.3%) Percentage of patients with hospitalization: Pharmacist-led intervention (22.3%) vs. usual care (25.5%)	Pharmacist-integrated CGM optimized therapy and reduced patients hospitalized during the study period.
DeBoest, 2025 [86]	RCT	Ambulatory care setting	Adults with T2D (n = 57)	rtCGM	Pharmacist intervention with rtCGMs	Pharmacist intervention with rtCGMs vs. standard of care (SOC)	HbA1c: 10.5 ± 1.09%	3 months	HbA1c: SOC: −2.05 ± 1.20% rtCGM: −2.94 ± 1.18%, *p* = 0.0165	Greater HbA1c reduction and more medication interventions with pharmacist use of rtCGM compared with pharmacist care alone

Abbreviations: CGM, continuous glucose monitoring; Pro CGM, professional continuous glucose monitoring; rtCGM, real-time continuous glucose monitoring; HbA1c, glycated hemoglobin; TIR, time in range; TAR, time above range; TBR, time below range; T1D, type 1 diabetes; T2D, type 2 diabetes; RPh, pharmacist; DSMES, diabetes self-management education and support; SMA, shared medical appointment; DMEC, diabetes management and education clinic; CPT, current procedural terminology; RCT, randomized controlled trial; SOC, standard of care; FQHC, federally qualified health center.

## Data Availability

No new data were created or analyzed in this study.

## Supplementary Materials

The following supporting information can be downloaded at: https://www.mdpi.com/article/10.3390/healthcare14081019/s1, Figure S1. Schematic representation of a CGM system and its operating principle; Figure S2. Evolutionary trajectory of CGM systems toward IoT-enabled smart diabetes management; Table S1. Classification of CGM modalities and representative devices. Table S2. Representative studies providing contextual background on CGM use in T1D; Table S3. Representative studies providing contextual background on CGM use in T2D; Table S4. Representative studies providing contextual background on CGM use in pregnancy and gestational diabetes.

## Abbreviations

The following abbreviations are used in this manuscript:

ADA	American Diabetes Association
AGP	Ambulatory glucose profile
AID	Automated insulin delivery
AI	Artificial intelligence
BGM	Blood glucose monitoring
CAGR	Compound annual growth rate
CGM	Continuous glucose monitoring
CMS	Centers for Medicare & Medicaid Services
CPT	Current Procedural Terminology
DME	Durable medical equipment
DM	Diabetes mellitus
DSME	Diabetes self-management education
EASD	European Association for the Study of Diabetes
EHR	Electronic health record
FHIR	Fast Healthcare Interoperability Resources
FQHC	Federally Qualified Health Center
GDM	Gestational diabetes mellitus
G-BA	Federal Joint Committee (Germany)
GMI	Glucose management indicator
GOx	Glucose oxidase
HbA1c	Hemoglobin A1c
H_2_O_2_	Hydrogen peroxide
HCL	Hybrid closed-loop
HTA	Health technology assessment
IoT	Internet of Things
isCGM	Intermittently scanned continuous glucose monitoring
NDSS	National Diabetes Services Scheme (Australia)
NHS	National Health Service (United Kingdom)
PGHD	Patient-generated health data
POC	Point of care
RCTs	Randomized controlled trials
rtCGM	Real-time continuous glucose monitoring
SSV	Statutory Health Insurance Physicians (Germany)
T1D	Type 1 diabetes
T2D	Type 2 diabetes
TBR	Time below range
TIR	Time in range
WHO	World Health Organization

## References

[1] Rooney M.R., He J.H., Salpea P., Genitsaridi I., Magliano D.J., Boyko E.J., Wallace A.S., Fang M., Selvin E. (2025). Global and Regional Prediabetes Prevalence: Updates for 2024 and Projections for 2050. Diabetes Care.

[2] Sun H., Saeedi P., Karuranga S., Pinkepank M., Ogurtsova K., Duncan B.B., Stein C., Basit A., Chan J.C.N., Mbanya J.C. (2022). IDF Diabetes Atlas: Global, regional and country-level diabetes prevalence estimates for 2021 and projections for 2045. Diabetes Res. Clin. Pract..

[3] Harding J.L., Pavkov M.E., Magliano D.J., Shaw J.E., Gregg E.W. (2019). Global trends in diabetes complications: A review of current evidence. Diabetologia.

[4] Polonsky W.H., Fisher L. (2013). Self-monitoring of blood glucose in noninsulin-using type 2 diabetic patients: Right answer, but wrong question: Self-monitoring of blood glucose can be clinically valuable for noninsulin users. Diabetes Care.

[5] Beck R.W., Riddlesworth T., Ruedy K., Ahmann A., Bergenstal R., Haller S., Kollman C., Kruger D., McGill J.B., Polonsky W. (2017). Effect of Continuous Glucose Monitoring on Glycemic Control in Adults with Type 1 Diabetes Using Insulin Injections: The DIAMOND Randomized Clinical Trial. JAMA.

[6] Contreras I., Vehi J. (2018). Artificial Intelligence for Diabetes Management and Decision Support: Literature Review. J. Med. Internet Res..

[7] Duckworth C., Guy M.J., Kumaran A., O’Kane A.A., Ayobi A., Chapman A., Marshall P., Boniface M. (2024). Explainable Machine Learning for Real-Time Hypoglycemia and Hyperglycemia Prediction and Personalized Control Recommendations. J. Diabetes Sci. Technol..

[8] Lee S., Do H. (2018). Comparison and Analysis of ISO/IEEE 11073, IHE PCD-01, and HL7 FHIR Messages for Personal Health Devices. Healthc. Inform. Res..

[9] Gee J.S., Rosario N., Garey K.W., Asias-Dinh B. (2025). Effectiveness of Continuous Glucose Monitoring in a Pharmacist-Run Collaborative Drug Therapy Management Service for Underserved Individuals With Diabetes: A Quasi-Experimental Study. Clin. Diabetes.

[10] Sherrill C.H., Houpt C.T., Dixon E.M., Richter S.J. (2020). Effect of Pharmacist-Driven Professional Continuous Glucose Monitoring in Adults with Uncontrolled Diabetes. J. Manag. Care Spec. Pharm..

[11] Peled O., Vitzrabin Y., Beit Ner E., Lazaryan M., Berlin M., Barchel D., Berkovitch M., Beer Y., Tamir E. (2023). Acceptance rate of clinical pharmacists’ recommendations-an ongoing journey for integration. Front. Pharmacol..

[12] Gabbay M.A.L., Rodacki M., Calliari L.E., Vianna A.G.D., Krakauer M., Pinto M.S., Reis J.S., Puñales M., Miranda L.G., Ramalho A.C. (2020). Time in range: A new parameter to evaluate blood glucose control in patients with diabetes. Diabetol. Metab. Syndr..

[13] Scuffi C. (2014). Interstitium versus Blood Equilibrium in Glucose Concentration and its Impact on Subcutaneous Continuous Glucose Monitoring Systems. Eur. Endocrinol..

[14] Boehm R., Donovan J., Sheth D., Durfor A., Roberts J., Isayeva I. (2019). In Vitro Sugar Interference Testing with Amperometric Glucose Oxidase Sensors. J. Diabetes Sci. Technol..

[15] Di Molfetta S., Rossi A., Assaloni R., Cherubini V., Consoli A., Di Bartolo P., Guardasole V., Laurenzi A., Lombardo F., Maffeis C. (2022). A guide for the use of LibreView digital diabetes platform in clinical practice: Expert paper of the Italian Working Group on Diabetes and Technology. Diabetes Res. Clin. Pract..

[16] Veluvali A., Dehghani Zahedani A., Hosseinian A., Aghaeepour N., McLaughlin T., Woodward M., DiTullio A., Hashemi N., Snyder M.P. (2025). Impact of digital health interventions on glycemic control and weight management. Npj Digit. Med..

[17] Suh M.K., Woodbridge J., Moin T., Lan M., Alshurafa N., Samy L., Mortazavi B., Ghasemzadeh H., Bui A., Ahmadi S. (2012). Dynamic Task Optimization in Remote Diabetes Monitoring Systems. 2012 IEEE Second International Conference on Healthcare Informatics, Imaging and Systems Biology, La Jolla, CA, USA, 27–28 September 2012.

[18] Longo R., Sperling S. (2019). Personal Versus Professional Continuous Glucose Monitoring: When to Use Which on Whom. Diabetes Spectr..

[19] American Diabetes Association Professional Practice Committee (2024). 7. Diabetes Technology: Standards of Care in Diabetes-2024. Diabetes Care.

[20] Christy A., Fernanda F., Insani W.N., Abdulah R. (2025). Pharmacist-Led Digital Health Interventions for Patients with Diabetes: A Systematic Review. J. Multidiscip. Healthc..

[21] Yoo J.H., Kim J.H. (2023). Advances in Continuous Glucose Monitoring and Integrated Devices for Management of Diabetes with Insulin-Based Therapy: Improvement in Glycemic Control. Diabetes Metab. J..

[22] Didyuk O., Econom N., Guardia A., Livingston K., Klueh U. (2021). Continuous Glucose Monitoring Devices: Past, Present, and Future Focus on the History and Evolution of Technological Innovation. J. Diabetes Sci. Technol..

[23] Almurashi A.M., Rodriguez E., Garg S.K. (2023). Emerging Diabetes Technologies: Continuous Glucose Monitors/Artificial Pancreases. J. Indian Inst. Sci..

[24] Anandhakrishnan A., Hussain S. (2024). Automating insulin delivery through pump and continuous glucose monitoring connectivity: Maximizing opportunities to improve outcomes. Diabetes Obes. Metab..

[25] Tarumi S., Takeuchi W., Chalkidis G., Rodriguez-Loya S., Kuwata J., Flynn M., Turner K.M., Sakaguchi F.H., Weir C., Kramer H. (2021). Leveraging Artificial Intelligence to Improve Chronic Disease Care: Methods and Application to Pharmacotherapy Decision Support for Type-2 Diabetes Mellitus. Methods Inf. Med..

[26] Shang T., Zhang J.Y., Bequette B.W., Raymond J.K., Coté G., Sherr J.L., Castle J., Pickup J., Pavlovic Y., Espinoza J. (2021). Diabetes Technology Meeting 2020. J. Diabetes Sci. Technol..

[27] van Beers C.A., DeVries J.H., Kleijer S.J., Smits M.M., Geelhoed-Duijvestijn P.H., Kramer M.H., Diamant M., Snoek F.J., Serné E.H. (2016). Continuous glucose monitoring for patients with type 1 diabetes and impaired awareness of hypoglycaemia (IN CONTROL): A randomised, open-label, crossover trial. Lancet Diabetes Endocrinol..

[28] Bolinder J., Antuna R., Geelhoed-Duijvestijn P., Kröger J., Weitgasser R. (2016). Novel glucose-sensing technology and hypoglycaemia in type 1 diabetes: A multicentre, non-masked, randomised controlled trial. Lancet.

[29] Lind M., Polonsky W., Hirsch I.B., Heise T., Bolinder J., Dahlqvist S., Schwarz E., Ólafsdóttir A.F., Frid A., Wedel H. (2017). Continuous Glucose Monitoring vs Conventional Therapy for Glycemic Control in Adults With Type 1 Diabetes Treated With Multiple Daily Insulin Injections: The GOLD Randomized Clinical Trial. JAMA.

[30] Heinemann L., Freckmann G., Ehrmann D., Faber-Heinemann G., Guerra S., Waldenmaier D., Hermanns N. (2018). Real-time continuous glucose monitoring in adults with type 1 diabetes and impaired hypoglycaemia awareness or severe hypoglycaemia treated with multiple daily insulin injections (HypoDE): A multicentre, randomised controlled trial. Lancet.

[31] Laffel L.M., Kanapka L.G., Beck R.W., Bergamo K., Clements M.A., Criego A., DeSalvo D.J., Goland R., Hood K., Liljenquist D. (2020). Effect of Continuous Glucose Monitoring on Glycemic Control in Adolescents and Young Adults With Type 1 Diabetes: A Randomized Clinical Trial. JAMA.

[32] Leelarathna L., Evans M.L., Neupane S., Rayman G., Lumley S., Cranston I., Narendran P., Barnard-Kelly K., Sutton C.J., Elliott R.A. (2022). Intermittently Scanned Continuous Glucose Monitoring for Type 1 Diabetes. N. Engl. J. Med..

[33] Bajaj M., McCoy R.G., Balapattabi K., Bannuru R.R., Bellini N.J., Bennett A.K., Beverly E.A., Briggs Early K., ChallaSivaKanaka S., Echouffo-Tcheugui J.B. (2025). 7. Diabetes Technology: Standards of Care in Diabetes—2026. Diabetes Care.

[34] Vigersky R.A., Fonda S.J., Chellappa M., Walker M.S., Ehrhardt N.M. (2012). Short- and long-term effects of real-time continuous glucose monitoring in patients with type 2 diabetes. Diabetes Care.

[35] Wada E., Onoue T., Kobayashi T., Handa T., Hayase A., Ito M., Furukawa M., Okuji T., Okada N., Iwama S. (2020). Flash glucose monitoring helps achieve better glycemic control than conventional self-monitoring of blood glucose in non-insulin-treated type 2 diabetes: A randomized controlled trial. BMJ Open Diabetes Res. Care.

[36] Aronson R., Brown R.E., Chu L., Bajaj H.S., Khandwala H., Abitbol A., Malakieh N., Goldenberg R. (2023). IMpact of flash glucose Monitoring in pEople with type 2 Diabetes Inadequately controlled with non-insulin Antihyperglycaemic ThErapy (IMMEDIATE): A randomized controlled trial. Diabetes Obes. Metab..

[37] Layne J.E., Jepson L.H., Carite A.M., Parkin C.G., Bergenstal R.M. (2024). Long-Term Improvements in Glycemic Control with Dexcom CGM Use in Adults with Noninsulin-Treated Type 2 Diabetes. Diabetes Technol. Ther..

[38] Ehrhardt N.M., Chellappa M., Walker M.S., Fonda S.J., Vigersky R.A. (2011). The effect of real-time continuous glucose monitoring on glycemic control in patients with type 2 diabetes mellitus. J. Diabetes Sci. Technol..

[39] Sierra J.A., Shah M., Gill M.S., Flores Z., Chawla H., Kaufman F.R., Vigersky R. (2018). Clinical and economic benefits of professional CGM among people with type 2 diabetes in the United States: Analysis of claims and lab data. J. Med. Econ..

[40] Grace T., Salyer J. (2022). Use of Real-Time Continuous Glucose Monitoring Improves Glycemic Control and Other Clinical Outcomes in Type 2 Diabetes Patients Treated with Less Intensive Therapy. Diabetes Technol. Ther..

[41] Lever C.S., Williman J.A., Boucsein A., Watson A., Sampson R.S., Sergel-Stringer O.T., Keesing C., Chepulis L., Wheeler B.J., de Bock M.I. (2024). Real time continuous glucose monitoring in high-risk people with insulin-requiring type 2 diabetes: A randomised controlled trial. Diabet. Med..

[42] Secher A.L., Ringholm L., Andersen H.U., Damm P., Mathiesen E.R. (2013). The effect of real-time continuous glucose monitoring in pregnant women with diabetes: A randomized controlled trial. Diabetes Care.

[43] Wei Q., Sun Z., Yang Y., Yu H., Ding H., Wang S. (2016). Effect of a CGMS and SMBG on Maternal and Neonatal Outcomes in Gestational Diabetes Mellitus: A Randomized Controlled Trial. Sci. Rep..

[44] Feig D.S., Donovan L.E., Corcoy R., Murphy K.E., Amiel S.A., Hunt K.F., Asztalos E., Barrett J.F.R., Sanchez J.J., de Leiva A. (2017). Continuous glucose monitoring in pregnant women with type 1 diabetes (CONCEPTT): A multicentre international randomised controlled trial. Lancet.

[45] Valent A.M., Rickert M., Pagan C.H., Ward L., Dunn E., Rincon M. (2025). Real-Time Continuous Glucose Monitoring in Pregnancies With Gestational Diabetes Mellitus: A Randomized Controlled Trial. Diabetes Care.

[46] Liu M., Chen T., Wang S., Li N., Liu D. (2025). To assess the impact of individualized strategy and continuous glucose monitoring on glycemic control and mental health in pregnant women with diabetes. Front. Endocrinol..

[47] Mordor Intelligence (2025). Continuous Glucose Monitoring Market Size & Share Analysis—Growth Trends & Forecasts (2025–2030).

[48] Aleppo G., Hirsch I.B., Parkin C.G., McGill J., Galindo R., Kruger D.F., Levy C.J., Forlenza G.P., Umpierrez G.E., Grunberger G. (2023). Coverage for Continuous Glucose Monitoring for Individuals with Type 2 Diabetes Treated with Nonintensive Therapies: An Evidence-Based Approach to Policymaking. Diabetes Technol. Ther..

[49] Ng S.M. (2023). NICE and NHS England leads the way to improve diabetes care with access to continuous glucose monitoring for people with type 1 diabetes. BMC Med..

[50] Schlüter S., Deiss D., Gehr B., Lange K., von Sengbusch S., Thomas A., Ziegler R., Freckmann G. (2024). Glucose Monitoring and Control Testing in Patients with Type 1 or Type 2 diabetes. Exp. Clin. Endocrinol. Diabetes.

[51] Santova A., de Bock M., Lanzinger S., Goldbloom E.B., Bratina N., Barcala C., Alhomaidah D., Pande A.R., Guness P.K., Dzivite-Krisane I. (2025). Global Inequities in Diabetes Technology and Insulin Access and Glycemic Outcomes. JAMA Netw. Open.

[52] Isaacs D., Bellini N.J., Biba U., Cai A., Close K.L. (2021). Health Care Disparities in Use of Continuous Glucose Monitoring. Diabetes Technol. Ther..

[53] Hall T., Warman M.K., Oser T., Filippi M.K., Manning B., Carroll J.K., Nease D.E., Staton E.W., Oser S. (2024). Clinician-Reported Barriers and Needs for Implementation of Continuous Glucose Monitoring. J. Am. Board Fam. Med..

[54] Wyckoff J.A., Lapolla A., Asias-Dinh B.D., Barbour L.A., Brown F.M., Catalano P.M., Corcoy R., Di Renzo G.C., Drobycki N., Kautzky-Willer A. (2025). Preexisting Diabetes and Pregnancy: An Endocrine Society and European Society of Endocrinology Joint Clinical Practice Guideline. J. Clin. Endocrinol. Metab..

[55] Woodward A., Walters K., Davies N., Nimmons D., Protheroe J., Chew-Graham C.A., Stevenson F., Armstrong M. (2024). Barriers and facilitators of self-management of diabetes amongst people experiencing socioeconomic deprivation: A systematic review and qualitative synthesis. Health Expect..

[56] Willis M., Nilsson A., Alshannaq H., Matuoka J., Norman G. (2025). The cost-effectiveness of real-time continuous glucose monitoring versus intermittently scanned continuous glucose monitoring in individuals with insulin-treated Type 2 diabetes mellitus in Canada. J. Comp. Eff. Res..

[57] Isitt J.J., Roze S., Sharland H., Cogswell G., Alshannaq H., Norman G.J., Lynch P.M. (2022). Cost-Effectiveness of a Real-Time Continuous Glucose Monitoring System Versus Self-Monitoring of Blood Glucose in People with Type 2 Diabetes on Insulin Therapy in the UK. Diabetes Ther..

[58] Stegbauer C., Falivena C., Moreno A., Hentschel A., Rosenmöller M., Heise T., Szecsenyi J., Schliess F. (2020). Costs and its drivers for diabetes mellitus type 2 patients in France and Germany: A systematic review of economic studies. BMC Health Serv. Res..

[59] Abbott (2022). Abbott’s FreeStyle^®^ Libre Is First and Only CGM System to Gain Expanded Reimbursement in Japan to Include All People with Diabetes Who Use Insulin.

[60] Kang S., Kang S.M., Choi J.H., Ko S.H., Koo B.K., Kwon H.S., Kim M.K., Kim S.Y., Kim S.K., Kim Y.E. (2025). 2025 Clinical Practice Guidelines for Diabetes Management in Korea: Recommendation of the Korean Diabetes Association. Diabetes Metab. J..

[61] Kim J.Y., Kim S., Kim J.H. (2025). Current Status of Continuous Glucose Monitoring Use in South Korean Type 1 Diabetes Mellitus Population–Pronounced Age-Related Disparities: Nationwide Cohort Study. Diabetes Metab. J..

[62] Lee M.-J., Seo B.-J., Cho J.-H. (2025). Expanding the Use of Continuous Glucose Monitoring in Type 2 Diabetes Mellitus: Impact on Glycemic Control and Metabolic Health. Life.

[63] Robles-Plaza M., Gómez-Peralta F., Bellido V., Ampudia-Blasco F.J., Carretero-Gómez J., Cebrián-Cuenca A.M., de la Cuadra-Grande A., Mezquita-Raya P. (2025). Cost-Utility Analysis of FreeStyle Libre Systems in People with Type 2 Diabetes Mellitus on Treatment with Basal Insulin and Poor Glycemic Control in Spain. Diabetes Ther..

[64] Ampudia-Blasco F.J., de la Cuadra-Grande A., Bellido V., Cebrián-Cuenca A.M., Mezquita-Raya P., Carretero-Gómez J., Hernández Martínez A.-M., Oyagüez I., Gómez-Peralta F. (2025). Cost Analysis of the FreeStyle Libre Systems in People with Type 2 Diabetes Mellitus on Basal Insulin with Poor Glycemic Control: A Spanish Perspective. Diabetes Technol. Ther..

[65] Lomax K.E., Taplin C.E., Abraham M.B., Smith G.J., Haynes A., Zomer E., Ellis K.L., Clapin H., Zoungas S., Jenkins A.J. (2023). Socioeconomic status and diabetes technology use in youth with type 1 diabetes: A comparison of two funding models. Front. Endocrinol..

[66] Vascimini A., Saba Y., Baskharoun F., Crooks K., Huynh V., Wasson S., Wright E., Bullers K., Franks R., Carris N.W. (2023). Pharmacist-driven continuous glucose monitoring in community and ambulatory care pharmacy practice: A scoping review. J. Am. Pharm. Assoc..

[67] Miller L., Woodyear J., Marciniak M.W., Rhodes L.A. (2024). Evaluation of a community-based pharmacy resident-led continuous glucose monitoring program within a family medicine clinic. J. Am. Pharm. Assoc..

[68] Aronson R., Abitbol A., Bajaj H.S., Cheng A.Y.Y., Christopoulos S., Harris S.B., Jain A.B., Goldenberg R.M. (2025). Continuous glucose monitoring in noninsulin-treated type 2 diabetes: A critical review of reported trials with an updated systematic review and meta-analysis of randomised controlled trials. Diabetes Obes. Metab..

[69] Beldon C., Rogers K., Johnson A., Schneider R., Stinson L., Frede S., Johnson K. (2024). Assessment of a community pharmacist remote monitoring service in patients using continuous glucose monitors. J. Am. Pharm. Assoc..

[70] Evers M., Draime J., Barhorst R. (2025). Impact of pharmacist-led continuous glucose monitoring program on reduction in A1c in an FQHC. JAPhA Pract. Innov..

[71] Thurston J., Li H., Rajan M., Baratt Y., Bradley A., Pelzman F. (2025). Pharmacist Integration to Support Continuous Glucose Monitoring Initiation: A Collaborative, Patient-Centered Approach. J. Pharm. Pract..

[72] King J., Keedy C., Crosby J., Little S., Thompson A., Hardin D., Pierce K. (2025). Evaluating Pharmacotherapy Optimization in Pharmacist-Led Management of Type 2 Diabetes Utilizing Continuous Glucose Monitors. J. Prim. Care Community Health.

[73] Mnatzaganian C., Bounthavong M., Abalos W., Chau T., Nwosu O., Yi A., Saunders I., Kelly P. (2025). Evaluation of pharmacist-led management of type 2 diabetes using personal continuous glucose monitors across a large tertiary academic health system. J. Am. Pharm. Assoc..

[74] Al AdAwi R.M., Stewart D., Ryan C., Tonna A.P. (2020). A systematic review of pharmacist input to metabolic syndrome screening, management and prevention. Int. J. Clin. Pharm..

[75] Sørensen M., Groven K.S., Gjelsvik B., Almendingen K., Garnweidner-Holme L. (2020). The roles of healthcare professionals in diabetes care: A qualitative study in Norwegian general practice. Scand. J. Prim. Health Care.

[76] Nigro S.C., Garwood C.L., Berlie H., Irons B., Longyhore D., McFarland M.S., Saseen J.J., Trewet C.B. (2014). Clinical pharmacists as key members of the patient-centered medical home: An opinion statement of the Ambulatory Care Practice and Research Network of the American College of Clinical Pharmacy. Pharmacotherapy.

[77] Bajaj M., Romeo G., Peters A., Pilla S., Bennett A., ChallaSivaKanaka S., Segal A., Rosas S., Beverly E., Echouf-fo-Tcheugui J. (2025). Comprehensive Medical Evaluation and Assessment of Comorbidities: Standards of Care in Dia-betes—2026. Diabetes Care.

[78] Pasour T., Sheehan L., Troyer M., Conger M., Carson P. (2024). Evaluation of a pharmacist-led personal continuous glucose monitor workflow to improve glycemic management in an internal medicine clinic. J. Am. Pharm. Assoc..

[79] Cellino C.A.K., Chen E.-L., Pawelek J., Daly C.J., Jacobs D.M., Prescott G.M. (2026). Pharmacist’s role in addressing barriers to continuous glucose monitoring within underserved communities. J. Am. Pharm. Assoc..

[80] Ballard L., York A.L., Skelley J.W., Sims M. (2024). Clinical and Financial Outcomes of a Pilot Pharmacist-Led Continuous Glucose Monitoring Clinic. Innov. Pharm..

[81] Van Dril E., Schumacher C. (2019). Impact of professional continuous glucose monitoring by clinical pharmacists in an ambulatory care setting. JACCP J. Am. Coll. Clin. Pharm..

[82] Ulrich H., Bowen M. (2021). The clinical utility of professional continuous glucose monitoring by pharmacists for patients with type 2 diabetes. J. Am. Pharm. Assoc..

[83] Simonyan A.R., Isaacs D., Lekic S., Blanchette J.E., Noe D., Galloway N.R. (2021). Continuous glucose monitoring shared medical appointments improve diabetes self-efficacy and hemoglobin A1C. JACCP J. Am. Coll. Clin. Pharm..

[84] Fantasia K.L., Stockman M.-C., Ju Z., Ortega P., Crable E.L., Drainoni M.-L., Walkey A.J., Bergstrom M., O’Brien K., Steenkamp D. (2021). Professional continuous glucose monitoring and endocrinology eConsult for adults with type 2 diabetes in primary care: Results of a clinical pilot program. J. Clin. Transl. Endocrinol..

[85] Powell J., Mulani S.R. (2024). Partnering for Better Health: Using Continuous Glucose Monitoring and Clinical Pharmacist Collaboration to Improve Glycemic Control in Underserved Patients With Type 2 Diabetes. Clin. Ther..

[86] DeBoest A., Holley C., Hickey M., Kallenberger M., John J., Baker M. (2025). Evaluating the addition of real-time continuous glucose monitors to pharmacist intervention on glycated hemoglobin. Am. J. Health-Syst. Pharm..

[87] Ghasemi Z., Mousa R., Peiravian F., Yousefi N. (2025). Cost-effectiveness analysis of a community pharmacist-based intervention to prevent cardiovascular complications in patients with type 2 diabetes in Iran. Cost Eff. Resour. Alloc..

[88] Wang Y., Yeo Q.Q., Ko Y. (2016). Economic evaluations of pharmacist-managed services in people with diabetes mellitus: A systematic review. Diabet. Med..

[89] Allaire J.C., Dennis C., Wright E.E., Edelman S.V., Masturzo A. (2025). CGM Adherence and Costs Improve with DME Channel Over Pharmacy. Clin. Diabetes.

[90] Romeo G.R., Caputo T., Stanescu I.W., Alkhaddo J.B. (2024). The Arduous Path Toward Equitable Access to Endocrinology Care. J. Endocr. Soc..

[91] Sawyer K., Saxon D., Zane R., Patel H., McDermott M., Singh V., Lawler H.M. (2025). A successful remote patient monitoring program for diabetes. Front. Endocrinol..

[92] Eiland L.A., Drincic A. (2022). Rural Telehealth Visits in the Management of Type 1 Diabetes. J. Diabetes Sci. Technol..

[93] Kavookjian J., Bzowyckyj A.S., DiNardo M.M., Kocurek B., Kolb L.E., Noe D., Ryan D., Saunders M.M., See M., Uelmen S. (2022). Current and Emerging Trends in Diabetes Care and Education: 2021 National Practice and Workforce Survey. Sci. Diabetes Self Manag. Care.

[94] Poudel A., Nissen L.M. (2016). Telepharmacy: A pharmacist’s perspective on the clinical benefits and challenges. Integr. Pharm. Res. Pract..

[95] Ballantyne P.J. (2007). The role of pharmacists in primary care. BMJ.

[96] Argueta A.S., Ng J.M., Zupa M.F. (2026). Barriers to use of continuous glucose monitoring among adults with type 2 diabetes. Diabetes Res. Clin. Pract..

[97] Oser T.K., Hall T.L., Dickinson L.M., Callen E., Carroll J.K., Nease D.E., Michaels L., Oser S.M. (2022). Continuous Glucose Monitoring in Primary Care: Understanding and Supporting Clinicians’ Use to Enhance Diabetes Care. Ann. Fam. Med..

[98] Vimalananda V.G., Kragen B., Leibowitz A.J., Qian S., Wormwood J., Linsky A.M., Underwood P., Conlin P.R., Kim B. (2025). Determinants of implementation of continuous glucose monitoring for patients with Insulin-Treated type 2 diabetes: A national survey of primary care providers. BMC Prim. Care.

[99] Knezevich E., Fuji K.T., Larson K., Muniz G. (2022). A Cross-Sectional Survey Study Examining the Provision of Continuous Glucose Monitoring Education in U.S. Doctor of Pharmacy Programs. Pharmacy.

[100] Hussain S., Polonsky W., Scibilia R., Glatzer T. (2025). Beyond the Trend Arrow: Potential Value of Artificial Intelligence–Supported Glucose Predictions for People with Type 1 Diabetes Using Continuous Glucose Monitoring Systems. Diabetes Technol. Ther..

[101] Herrero P., Andorrà M., Babion N., Bos H., Koehler M., Klopfenstein Y., Leppäaho E., Lustenberger P., Peak A., Ringemann C. (2024). Enhancing the Capabilities of Continuous Glucose Monitoring With a Predictive App. J. Diabetes Sci. Technol..

[102] Ji C., Jiang T., Liu L., Zhang J., You L. (2025). Continuous glucose monitoring combined with artificial intelligence: Redefining the pathway for prediabetes management. Front. Endocrinol..

[103] Al-Taie A., Hafida M., Abdulsattar M., El Mahmoud R. (2026). Scoping insights into artificial intelligence-driven treatment of diabetes mellitus in clinical practice. Egypt. J. Intern. Med..

