# Supplementary material for: Integrating Pharmacists into CGM-Enabled Digital Diabetes Care: Advancing Personalized and Data-Driven Management
Source: Healthcare (Basel). 2026 Apr 13;14(8):1019. doi: 10.3390/healthcare14081019 (PMC13115816; doi:10.3390/healthcare14081019)
Supplement: Supplementary file 1 [file healthcare-14-01019-s001.zip › healthcare-4222749-supplementary.pdf]

Supplementary Information

Integrating Pharmacists into CGM-Enabled Digital Diabetes Care: Advancing Personalized and Data-Driven Management

Xiaoxiao Chen [1,†], Gyeong Eon Kim [2,†], Nam Ah Kim [2,*], and Kwang Joon Kim [1,*]

1 Affiliation 1; College of Pharmacy, Chonnam National University, Gwangju, Republic of Korea
2 Affiliation 2; College of Pharmacy, Mokpo National University, Muan, Republic of Korea
* Correspondence:
Kwang Joon Kim, RPh, Ph.D., College of Pharmacy, Chonnam National University,
77 Yongbong-ro, Buk-gu, Gwangju 61186, Republic of Korea;
kjkim0901@jnu.ac.kr (K.J.K.); Tel.: +82-62-530-2929.

Nam Ah Kim, RPh, Ph.D., College of Pharmacy, Mokpo National University,
Muan, Jeollanam-do 58554, Republic of Korea;
namahk87@mnu.ac.kr (N.A.K.); Tel.: +82-61-450-2685.

Table S1. Classification of CGM modalities and representative devices.

Modality	Definition	Key Characteristics	Representative Devices
rtCGM	Real-time continuous glucose monitoring automatically transmitted in real time	Provides real-time glucose readings without user scanning; supports alerts and remote data sharing	Libre 2 Plus and Libre 3 Plus, Dexcom G6 and G7, Eversense 365, Guardian 4, Simplera
isCGM	Intermittently scanned continuous glucose monitoring requiring user-initiated scanning for glucose readings	Glucose data are accessed via user-initiated scanning; newer versions may support optional real-time transmission	FreeStyle Libre, FreeStyle Libre 2, FreeStyle Libre 3
Pro CGM	Professional-use continuous glucose monitoring used under healthcare professional supervision	Typically clinic-based or blinded; data are retrospectively reviewed and used for clinical decision-making	Dexcom G6 Pro, Abbott FreeStyle Libre Pro

CGM modalities were classified as real-time CGM (rtCGM), intermittently scanned CGM (isCGM), and professional CGM (Pro CGM) based on their mode of glucose data acquisition and transmission, in accordance with current clinical practice guidelines and consensus reports from the American Diabetes Association (ADA)[1].

Table S2. Representative studies illustrating CGM use in T1D

Clinical Setting	Study (First author, year)	Study design (Country)	Population (n)	CGM type / comparator	Key findings
T1D	Beers et al., 2016 (IN CONTROL) [2]	RCT (Netherlands)	Adults with T1D and impaired hypoglycemia awareness (n = 52)	rtCGM (Medtronic Paradigm Veo) vs BGM	Normoglycemia +9.6%; hyperglycemia and hypoglycemia: -4.7% ($p < 0.0001$)
	Bolinder et al., 2016[3]	RCT (Europe)	Adult with well-controlled T1D; HbA1c ≤ 7.5% (n = 328)	isCGM (FreeStyle Libre) vs BGM	Hypoglycemia: -1.24 h/day ($p < 0.0001$)
	Lind et al., 2017 (GOLD) [4]	RCT (Sweden)	Adults with T1D on MDI (n = 161)	rtCGM (Dexcom G4) vs BGM	HbA1c: -0.43% ($p < 0.001$)
	Beck et al., 2017 (DIAMOND)[5]	RCT (USA)	Adults with T1D on MDI (n = 158)	rtCGM (Dexcom G4) vs BGM	HbA1c: -0.6% (24 weeks; $p < 0.001$)
	Heinemann et al., 2018 (HypoDE) [6]	RCT (Germany)	Adults with T1D and recurrent hypoglycemia (n = 149)	rtCGM (Dexcom G5 Mobile) vs BGM	Hypoglycemic events: -72% ($p < 0.0001$).
	Laffel et al. 2020 [7]	RCT (USA)	Adolescents and young adults with T1D on MDI (n = 153)	rtCGM (Dexcom G5 Mobile) vs BGM	HbA1c: -0.37% (26 weeks; $p = 0.01$)
	Leelarathna et al., 2022[8]	RCT (UK)	Adults with T1D; HbA1c 7.5-11.0% (n = 156)	isCGM (FreeStyle Libre) vs BGM	HbA1c: -0.5% (24 weeks; $p < 0.001$)

Abbreviations: BGM, blood glucose monitoring; CGM, continuous glucose monitoring; rtCGM, real-time CGM; isCGM, intermittently scanned CGM; HbA1c, hemoglobin A1c; MDI, multiple daily insulin injections; RCT, randomized controlled trial; T1D, type 1 diabetes.

Table S3. Representative studies illustrating CGM use in T2D

Clinical setting	Study (first author, year)	Study design (Country)	Population (n)	CGM type / comparator	Key findings
Non-Insulin Treated T2D	Vigersky et al., 2012[9]	RCT (USA)	Adults with T2D not on prandial insulin (n = 100)	rtCGM (Dexcom SEVEN) vs BGM	HbA1c: −0.5% to −0.7% across follow-up (p = 0.04)
	Wada et al., 2020[10]	RCT (Japan)	Adults with noninsulin-treated T2D (n = 100)	isCGM (FreeStyle Libre) vs BGM	HbA1c: -0.22% (p = 0.022); TIR: +2.36 h (p < 0.01)
	Aronson et al., 2023[11]	RCT (Canada)	Adults with noninsulin-treated T2D (n = 116)	isCGM (FreeStyle Libre 2) + DSME or DSME alone	HbA1c: -0.3% (p = 0.048); TIR: +9.9% (p < 0.01)
	Layne et al., 2024 [12]	Retrospective cohort (USA)	Adults with noninsulin-treated T2D (n = 3,840)	rtCGM (Dexcom G6) vs BGM	TIR: +17.3% (p < 0.001); GMI: -0.5% (p < 0.001); TITR: +16.4% (p < 0.001)
Insulin Treated T2D	Ehrhardt et al., 2011[13]	RCT (USA)	Adults with T2D (n = 50)	rtCGM (Dexcom SEVEN Plus) vs BGM	HbA1c: −0.95% vs −0.46%
	Sierra et al., 2018[14]	Claims-based (USA)	Adults with T2D (n = 11,354)	Pro CGM (FreeStyle Libre Pro) vs BGM	HbA1c: -0.44% (p < 0.001)
	Grace, et al. 2022[15]	Retrospective cohort (USA)	Adults with T2D treated with intensive insulin therapy (n=38)	rtCGM (Dexcom G6) vs BGM	HbA1c: −3.0% (p < 0.001); TIR: +15.2% (p < 0.001)
	Claire S Lever et al. 2024 [16]	RCT (New Zealand)	Adults with insulin-treated T2D (n=67)	rtCGM (Dexcom G6) vs BGM	TIR: +13% (p=0.007); HbA1c: -1.4% (p<0.001)

Abbreviations: BGM, blood glucose monitoring; CGM, continuous glucose monitoring; rtCGM, real-time CGM; isCGM, intermittently scanned CGM; DSME, diabetes self-management education; HbA1c, glycated hemoglobin; TIR, time in range; GMI, glucose management indicator; RCT, randomized controlled trial; T2D, type 2 diabetes.

Table S4. Representative studies illustrating CGM use in pregnancy and gestational diabetes (GDM)

Clinical setting	Study (first author, year)	Study design / country	Population and sample size	CGM type / comparator	Key findings
Pregnancy and GDM	Secher et al., 2013 [17]	RCT (Europe)	Pregnancy women with T1D or T2D (n = 123)	rtCGM (Medtronic Guardian) vs BGM	No significant difference in HbA1c; no significant difference in large-for-gestational-age infants
	Wei et al., 2016[18]	RCT (China)	Women with GDM in gestational weeks 24-28 (n = 106)	Pro CGM (Medtronic CGMS Gold) vs BGM	No significant difference in HbA1c; improved maternal metrics
	Feig et al., 2017 (CONCEPTT) [19]	Multicenter RCT	Pregnant women with T1D (n = 325)	rtCGM (Dexcom G4) vs BGM	HbA1c: -0.19% (p = 0.0207); TIR: +7% (p = 0.0034); hyperglycemic: -5% (p = 0.0279);
	Valent et al., 2025[20]	RCT (USA)	Pregnancies (>20 weeks of gestation) with GDM (n = 111)	rtCGM (Dexcom G6) vs CBG	TIR: 5% (p = 0.027)
	Liu et al., 2025[21]	RCT (China)	Pregnant women with T2D (n = 80)	isCGM (FreeStyle Libre 3) vs BGM	Improved glycemic control and patient-reported outcomes

Abbreviations: BGM, blood glucose monitoring; CGM, continuous glucose monitoring; rtCGM, real-time CGM; HbA1c, glycated hemoglobin; GDM, gestational diabetes mellitus; TIR, time in range; RCT, randomized controlled trial; T1D, type 1 diabetes; T2D, type 2 diabetes.

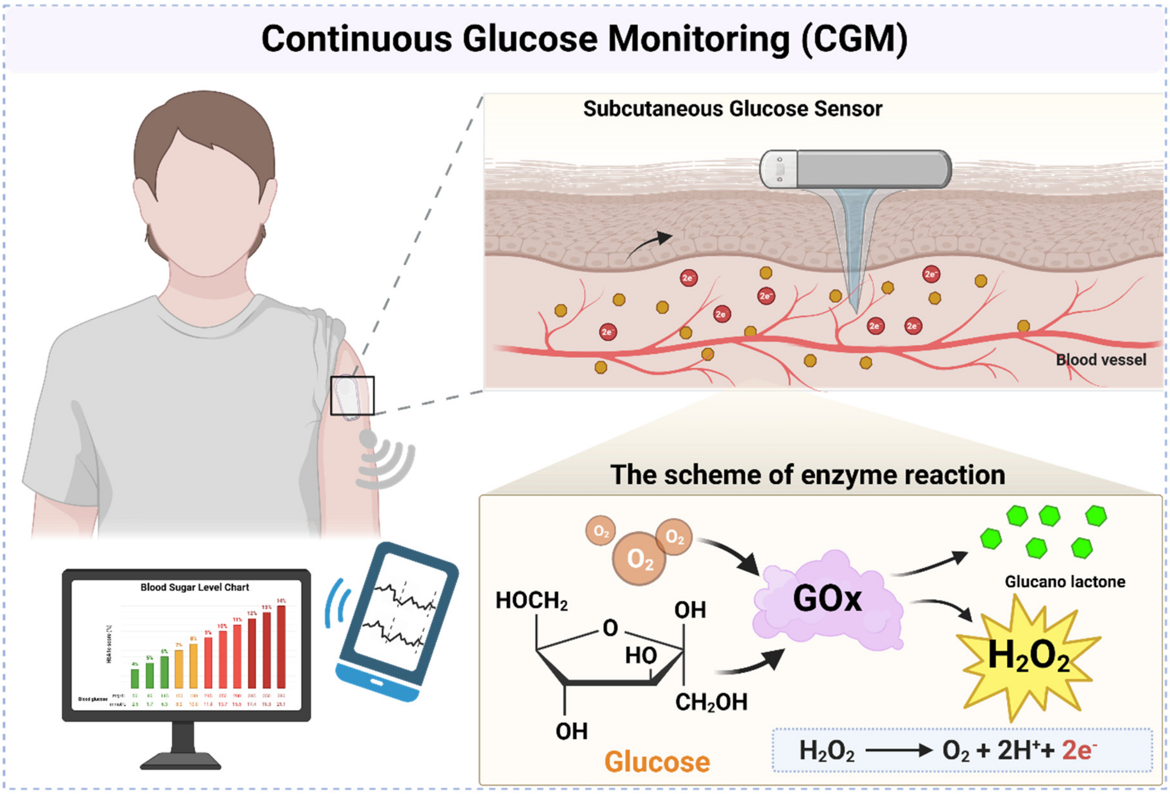

Figure S1. Schematic representation of a CGM system and its operating principle. Interstitial glucose diffuses into the subcutaneous sensor and is oxidized by glucose oxidase, producing gluconolactone and hydrogen peroxide (H_2O_2). The electrochemical detection of H_2O_2 generates a current proportional to glucose concentration, which is converted into real-time glucose readings.

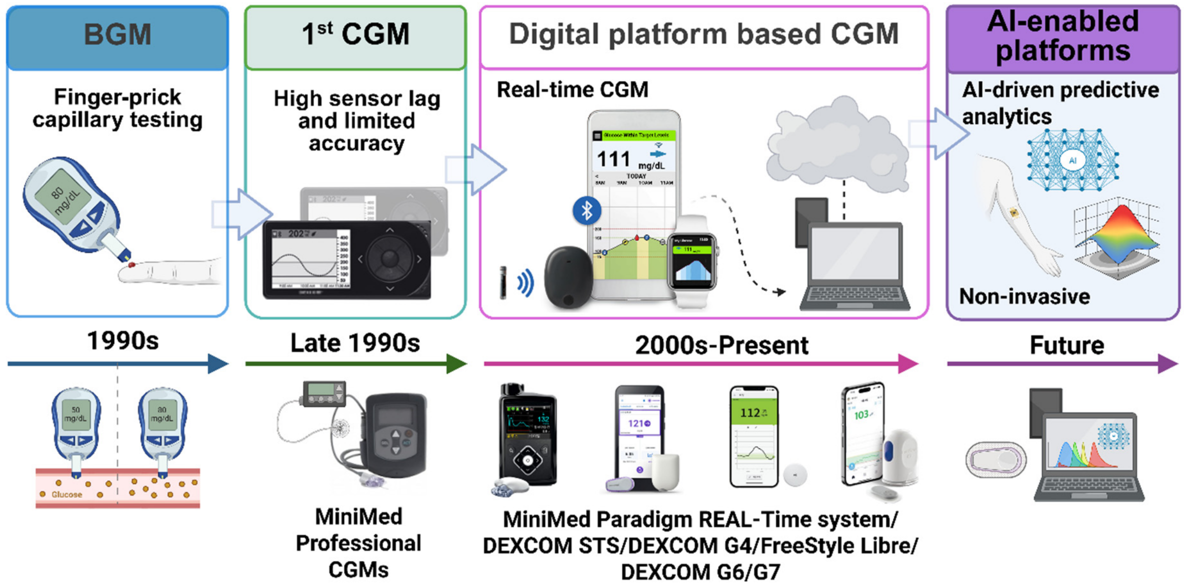

Figure S2. Evolutionary trajectory of CGM systems toward Internet of Things (IoT)-enabled smart diabetes management. Glucose monitoring has progressed from BGM to early-generation CGM with limited accuracy, followed by real-time CGM integrated with digital platforms, and most recently to AI-enabled systems supporting predictive analytics and personalized, data-driven diabetes management. IoT-enabled refers to Internet of Things-based CGM systems that enable real-time data transmission, cloud-based integration, and remote data access.

References

1. Diabetes*, A. D. A. P. P. C. f., 7. Diabetes Technology: Standards of Care in Diabetes—2026. *Diabetes Care* **2025,** *49* (Supplement_1), S150-S165.

2. van Beers, C. A.; DeVries, J. H.; Kleijer, S. J.; Smits, M. M.; Geelhoed-Duijvestijn, P. H.; Kramer, M. H.; Diamant, M.; Snoek, F. J.; Serné, E. H., Continuous glucose monitoring for patients with type 1 diabetes and impaired awareness of hypoglycaemia (IN CONTROL): a randomised, open-label, crossover trial. *Lancet Diabetes Endocrinol* **2016,** *4* (11), 893-902.

3. Bolinder, J.; Antuna, R.; Geelhoed-Duijvestijn, P.; Kröger, J.; Weitgasser, R., Novel glucose-sensing technology and hypoglycaemia in type 1 diabetes: a multicentre, non-masked, randomised controlled trial. *Lancet* **2016,** *388* (10057), 2254-2263.

4. Lind, M.; Polonsky, W.; Hirsch, I. B.; Heise, T.; Bolinder, J.; Dahlqvist, S.; Schwarz, E.; Ólafsdóttir, A. F.; Frid, A.; Wedel, H.; Ahlén, E.; Nyström, T.; Hellman, J., Continuous Glucose Monitoring vs Conventional Therapy for Glycemic Control in Adults With Type 1 Diabetes Treated With Multiple Daily Insulin Injections: The GOLD Randomized Clinical Trial. *Jama* **2017,** *317* (4), 379-387.

5. Beck, R. W.; Riddlesworth, T.; Ruedy, K.; Ahmann, A.; Bergenstal, R.; Haller, S.; Kollman, C.; Kruger, D.; McGill, J. B.; Polonsky, W.; Toschi, E.; Wolpert, H.; Price, D., Effect of Continuous Glucose Monitoring on Glycemic Control in Adults With Type 1 Diabetes Using Insulin Injections: The DIAMOND Randomized Clinical Trial. *Jama* **2017,** *317* (4), 371-378.

6. Heinemann, L.; Freckmann, G.; Ehrmann, D.; Faber-Heinemann, G.; Guerra, S.; Waldenmaier, D.; Hermanns, N., Real-time continuous glucose monitoring in adults with type 1 diabetes and impaired hypoglycaemia

awareness or severe hypoglycaemia treated with multiple daily insulin injections (HypoDE): a multicentre, randomised controlled trial. *Lancet* **2018,** *391* (10128), 1367-1377.

7. Laffel, L. M.; Kanapka, L. G.; Beck, R. W.; Bergamo, K.; Clements, M. A.; Criego, A.; DeSalvo, D. J.; Goland, R.; Hood, K.; Liljenquist, D.; Messer, L. H.; Monzavi, R.; Mouse, T. J.; Prahalad, P.; Sherr, J.; Simmons, J. H.; Wadwa, R. P.; Weinstock, R. S.; Willi, S. M.; Miller, K. M., Effect of Continuous Glucose Monitoring on Glycemic Control in Adolescents and Young Adults With Type 1 Diabetes: A Randomized Clinical Trial. *Jama* **2020,** *323* (23), 2388-2396.

8. Leelarathna, L.; Evans, M. L.; Neupane, S.; Rayman, G.; Lumley, S.; Cranston, I.; Narendran, P.; Barnard-Kelly, K.; Sutton, C. J.; Elliott, R. A.; Taxiarchi, V. P.; Gkountouras, G.; Burns, M.; Mubita, W.; Kanumilli, N.; Camm, M.; Thabit, H.; Wilmot, E. G., Intermittently Scanned Continuous Glucose Monitoring for Type 1 Diabetes. *New England Journal of Medicine* **2022,** *387* (16), 1477-1487.

9. Vigersky, R. A.; Fonda, S. J.; Chellappa, M.; Walker, M. S.; Ehrhardt, N. M., Short- and long-term effects of real-time continuous glucose monitoring in patients with type 2 diabetes. *Diabetes Care* **2012,** *35* (1), 32-8.

10. Wada, E.; Onoue, T.; Kobayashi, T.; Handa, T.; Hayase, A.; Ito, M.; Furukawa, M.; Okuji, T.; Okada, N.; Iwama, S.; Sugiyama, M.; Tsunekawa, T.; Takagi, H.; Hagiwara, D.; Ito, Y.; Suga, H.; Banno, R.; Kuwatsuka, Y.; Ando, M.; Goto, M.; Arima, H., Flash glucose monitoring helps achieve better glycemic control than conventional self-monitoring of blood glucose in non-insulin-treated type 2 diabetes: a randomized controlled trial. *BMJ Open Diabetes Res Care* **2020,** *8* (1).

11. Aronson, R.; Brown, R. E.; Chu, L.; Bajaj, H. S.; Khandwala, H.; Abitbol, A.; Malakieh, N.; Goldenberg, R., IMpact of flash glucose Monitoring in pEople with type 2 Diabetes Inadequately controlled with non-insulin Antihyperglycaemic ThErapy (IMMEDIATE): A randomized controlled trial. *Diabetes Obes Metab* **2023,** *25* (4), 1024-1031.

12. Layne, J. E.; Jepson, L. H.; Carite, A. M.; Parkin, C. G.; Bergenstal, R. M., Long-Term Improvements in Glycemic Control with Dexcom CGM Use in Adults with Noninsulin-Treated Type 2 Diabetes. *Diabetes Technol Ther* **2024,** *26* (12), 925-931.

13. Ehrhardt, N. M.; Chellappa, M.; Walker, M. S.; Fonda, S. J.; Vigersky, R. A., The effect of real-time continuous glucose monitoring on glycemic control in patients with type 2 diabetes mellitus. *J Diabetes Sci Technol* **2011,** *5* (3), 668-75.

14. Sierra, J. A.; Shah, M.; Gill, M. S.; Flores, Z.; Chawla, H.; Kaufman, F. R.; Vigersky, R., Clinical and economic benefits of professional CGM among people with type 2 diabetes in the United States: analysis of claims and lab data. *J Med Econ* **2018,** *21* (3), 225-230.

15. Grace, T.; Salyer, J., Use of Real-Time Continuous Glucose Monitoring Improves Glycemic Control and Other Clinical Outcomes in Type 2 Diabetes Patients Treated with Less Intensive Therapy. *Diabetes Technol Ther* **2022,** *24* (1), 26-31.

16. Lever, C. S.; Williman, J. A.; Boucsein, A.; Watson, A.; Sampson, R. S.; Sergel-Stringer, O. T.; Keesing, C.; Chepulis, L.; Wheeler, B. J.; de Bock, M. I.; Paul, R. G., Real time continuous glucose monitoring in high-risk people with insulin-requiring type 2 diabetes: A randomised controlled trial. *Diabet Med* **2024,** *41* (8), e15348.

17. Secher, A. L.; Ringholm, L.; Andersen, H. U.; Damm, P.; Mathiesen, E. R., The effect of real-time continuous glucose monitoring in pregnant women with diabetes: a randomized controlled trial. *Diabetes Care* **2013,** *36* (7), 1877-83.

18. Wei, Q.; Sun, Z.; Yang, Y.; Yu, H.; Ding, H.; Wang, S., Effect of a CGMS and SMBG on Maternal and Neonatal Outcomes in Gestational Diabetes Mellitus: a Randomized Controlled Trial. *Scientific Reports* **2016,** *6* (1), 19920.

19. Feig, D. S.; Donovan, L. E.; Corcoy, R.; Murphy, K. E.; Amiel, S. A.; Hunt, K. F.; Asztalos, E.; Barrett, J. F. R.; Sanchez, J. J.; de Leiva, A.; Hod, M.; Jovanovic, L.; Keely, E.; McManus, R.; Hutton, E. K.; Meek, C. L.;

Stewart, Z. A.; Wysocki, T.; O'Brien, R.; Ruedy, K.; Kollman, C.; Tomlinson, G.; Murphy, H. R., Continuous glucose monitoring in pregnant women with type 1 diabetes (CONCEPTT): a multicentre international randomised controlled trial. *Lancet* **2017,** *390* (10110), 2347-2359.

20. Valent, A. M.; Rickert, M.; Pagan, C. H.; Ward, L.; Dunn, E.; Rincon, M., Real-Time Continuous Glucose Monitoring in Pregnancies With Gestational Diabetes Mellitus: A Randomized Controlled Trial. *Diabetes Care* **2025,** *48* (9), 1581-1588.

21. Liu, M.; Chen, T.; Wang, S.; Li, N.; Liu, D., To assess the impact of individualized strategy and continuous glucose monitoring on glycemic control and mental health in pregnant women with diabetes. *Front Endocrinol (Lausanne)* **2025,** *16*, 1470473.
